# Spidroins under the Influence of Alcohol: Effect of Ethanol on Secondary Structure and Molecular Level Solvation of Silk-Like Proteins

**DOI:** 10.1021/acs.biomac.3c00637

**Published:** 2023-11-29

**Authors:** Dmitry A. Tolmachev, Maaria Malkamäki, Markus B. Linder, Maria Sammalkorpi

**Affiliations:** † Department of Chemistry and Materials Science, Aalto University, P.O. Box 16100, FI-00076 Aalto, Finland; ‡ Department of Bioproducts and Biosystems, Aalto University, P.O. Box 16100, FI-00076 Aalto, Finland; § Academy of Finland Center of Excellence in Life-Inspired Hybrid Materials (LIBER), Aalto University, P.O. Box 16100, FI-00076 Aalto, Finland

## Abstract

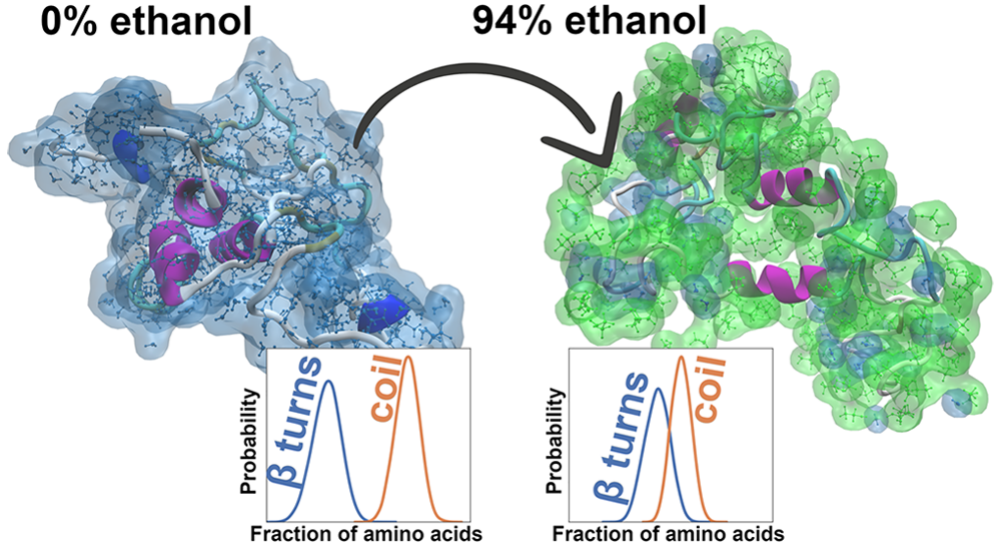

Future sustainable materials based on designer biomolecules require control of the solution assembly, but also interfacial interactions. Alcohol treatments of protein materials are an accessible means to this, making understanding of the process at the molecular level of seminal importance. We focus here on the influence of ethanol on spidroins, the main proteins of silk. By large-scale atomistically detailed molecular dynamics (MD) simulations and interconnected experiments, we characterize the protein aggregation, secondary structure changes, molecular level origins of them, and solvation environment changes for the proteins, as induced by ethanol as a solvation additive. The MD and circular dichoroism (CD) findings jointly show that ethanol promotes ordered structure in the protein molecules, leading to an increase of helix content and turns but also increased aggregation, as revealed by dynamic light scattering (DLS) and light microscopy. The structural changes correlate at the molecular level with increased intramolecular hydrogen bonding. The simulations reveal that polar amino acids, such as glutamine and serine, are most influenced by ethanol, whereas glycine residues are most prone to be involved in the ethanol-induced secondary structure changes. Furthermore, ethanol engages in interactions with the hydrophobic alanine-rich regions of the spidroin, significantly decreasing the hydrophobic interactions of the protein with itself and its surroundings. The protein solutes also change the microstructure of water/ethanol mixtures, essentially decreasing the level of larger local clustering. Overall, the work presents a systematic characterization of ethanol effects on a widely used, common protein type, spidroins, and generalizes the findings to other intrinsically disordered proteins by pinpointing the general features of the response. The results can aid in designing effective alcohol treatments for proteins, but also enable design and tuning of protein material properties by a relatively controllable solvation handle, the addition of ethanol.

## Introduction

Spidroins are the main proteins in spider silk.^1^ Spider silk is an exceptional biomaterial with a unique set of properties, such as a broad range of mechanical properties,^2^ biocompatibility,^3,4^ biodegradability,^5,6^ and high thermal conductivity.^7^ Together with emerging developments of large-scale synthetic biotechnology production of silk proteins,^8−11^ these properties make silk protein-based materials a fascinating prospect for material solutions in various application fields, such as tissue engineering, wound dressings, drug delivery, intelligent health monitoring, food packaging, and the textile industry.^12−15^

The material properties of silk proteins are determined by their supramolecular, secondary, and amino acid sequence. From a chemical point of view, silk proteins are triblock biopolymers with globular terminal units separated by an intrinsically disordered middle part consisting of repetitive domains, which contain hydrophilic glycine-rich and hydrophobic alanine-rich regions.^16−18^ The elevated content of glycine in the hydrophilic regions leads to a large conformational variation and structural disorder in these regions. The alanine-rich sequences tend to form α-helices in solution and β-sheets in fiber.^19−21^ The excellent mechanical properties of silk proteins rise from and are supported by the tendency of the proteins to assemble and form a network in which crystalline β-sheets stabilized by intermolecular H-bonding form a structural linkage as nodes. More generally, the secondary structure is a determining factor for, e.g., mechanical^22^ and optical properties,^23^ thermal stability,^24^ biodegradation rate,^25^ and water solubility^26^ of silk materials.

A highly useful tuning potential for the properties of silk protein-based materials opens by the secondary structure of silk protein fiber being sensitive to the environment. For example, the β-sheet content in the fiber can be increased by mechanical stretching^27,28^ or by organic solvent additives (methanol or ethanol).^23,29−33^ Actually, alcohol treatment is one of the most common steps in preparing materials from silk proteins. Its target is controlling the silk protein material structure and properties but also, simultaneously, serves as a sterilization method. Moreover, alcohol treatment is a common approach for the fixation of proteins on surfaces and inducing coagulation by changing the solubility of the protein.^34−36^

Although the influence of alcohol on the secondary structure of silk proteins is well-known, alcohol treatments are extremely common, and the influence of alcohol on the proteins has been studied for more than 20 years, most of the existing research work is devoted to studying the effect of alcohol on the structure of silk fibers and on the surfaces of silk protein materials.^23,29−33^ The number of works devoted to the investigation of the effect of alcohol on the secondary structure of dissolved spidroins is very small. This is surprising, as the solution secondary structure forms the basis of fibril assembly and surface interactions. In this context, it is important to note the study of Dicko et al.^37^ on the secondary structure of spidroin proteins showing the increase of the ordered structure of the dissolved spidroin with the addition of methanol. However, most studies concentrate on the effects at the macroscale, with a limited or a lack of discussion of the effects occurring at the molecular level. Molecular level understanding of the effect of alcohol would help to predict the amino acid sequence-dependent changes in the effects of solvent treatments such as alcohol treatment here.

Resolving the molecular level effects of alcohol and the dependencies of the secondary structure changes in the protein experimentally remains challenging due to several processes taking place when alcohol is added to a spidroin protein solution. In addition to the effect of alcohol on the secondary structure, ethanol can also provoke aggregation of the silk proteins. A very effective way to distinguish these effects and their influence on the proteins is to perform molecular dynamics (MD) simulations in atomistic detail. This approach allows studying a model of the system while considering the system variables separately and analyzing the effects as independent contributions. Atomistic MD modeling is a well-established method for studying proteins in solution^38,39^ but also polymers,^40,41^ which are very sensitive in their conformations to their solvation environment. In addition, MD allows direct observation of the molecular processes and distinguishing the role of each amino acid in the interactions of the proteins with the solution. Here, we target the alcohol-induced secondary structure and solvation changes of spidroins by MD simulations, supporting the simulation findings with the experimental circular dichroism (CD) characterization of the proteins.

Previously, atomistic MD simulations have been used to study the effect of alcohol on various proteins.^42−49^ The effect of alcohol can differ depending on the initial secondary and tertiary structures of the protein. Alcohol can denature a globular protein and disturb intraprotein hydrogen bond network due to hydrophobic interaction with ethanol.^50^ It can induce hydrogen bonding and provoke protein aggregation.^51^ However, ethanol added to solutions with proteins with a large amount of α-helices can lead to the stabilization of the structure of the protein and prevent its aggregation.^52^

To the best of our knowledge, studies devoted to the molecular level effects of alcohol on the secondary structure of individual spidroin molecules and their changes due to alcohol solvation have not yet been carried out. Simulation studies devoted to other aspects of silk materials are well discussed in the recent review of Barreiro et al.^53^ Many simulation studies focus on the investigation of molecular mechanisms behind the mechanical response of silk materials.^54−56^ Closest to our current study is the spider silk protein spidroin-1 structural investigation performed by Santos-Pint et al.^57^ However, the size of the examined protein was prohibitive due to extended duration simulations in the study, and the MD simulations were mainly used to obtain 3D silk protein models corresponding to experimental study setups.^57^

Besides the influence of alcohol on silk proteins, the effect of the proteins on alcohol/water mixtures is also important both fundamentally and for biotechnology processes. An alcohol/water mixture is well-known to have a heterogeneous structure at the microscale,^58−60^ representing two penetrating phases with large and small water concentrations. Adding a polymer to the solution locally changes this structure, increasing the phase separation or mixing the two phases, depending on the interaction between the polymer and the solvent molecules.

Altogether, understanding the influence of alcohol on the secondary structure of the proteins and also the effect of the proteins on the alcohol/water mixture aids controlled development of silk protein-based materials. Here, we combine detailed molecular modeling with an experimental characterization to extract both the overall influence of ethanol on the protein and protein on the ethanol/water mixture, but also derive the generalization guidelines for amino acid-specific responses and discuss the shift of inter/intramolecular interactions balanced with the addition of ethanol. The significance of the work is that the molecular level guidelines and understanding of the effect of the widely common solvent additive ethanol provide both fundamental and also processing level guidelines for protein materials. Such information is needed for gaining control of the solution assembly and interfacial interactions of protein-based materials in targeting future sustainable materials. Here, the effect is examined and mapped for a spidroin, but the amino acid level generalizations allow extending them to other proteins, and additionally, the demonstrated protocol can be expanded to other proteins.

## Materials and Methods

### Materials and Experimental Characterization

A recombinant silk protein with a general 3-block architecture, having a repetitive intrinsically disordered midblock and two terminal blocks of globular proteins, one at each end of the polymer, was used in the experiment. The midblock was an engineered version of the native ADF3 dragline silk sequence from *A. diadematus*,^61^ called AQ12 or eADF3, consisting of 12 repeating consensus sequences.^62^ The terminal blocks were a protein called cellulose binding module (CBM) from the *Clostridium thermocellum* cellulosome.^63^ Cloning, expression, and purification of the CBM-AQ12-CBM were carried out as described in our earlier study.^64^

Determination of the changes in the secondary structure of the protein was conducted by using a circular dichroism (CD) detector (Jasco J-1500–150ST). Ten accumulated scans for each sample with baseline correction were measured over wavelengths from 260 to 190 nm, with 1 nm resolution at 20 °C. High tension (HT) spectra were collected at the same time as the CD spectra to further monitor the quality of the CD data. Data were collected using a 1 mm path-length quartz cuvette (Hellma). The CD spectra are represented in molar ellipticity [*θ*] (deg·cm^2^·dmol^–1^), according to
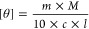
1where *m* is the CD signal (millidegrees), *M* is the average molecular weight (g/mol), *c* is the protein concentration (g/L), and *l* is the path length (cm).

The measured samples had the same initial protein concentration of 0.27 mg/mL, measured by a UV/vis spectrometer (Varian Cary 50) at 280 nm. The solvent was varied in samples with different concentrations of ethanol/reverse osmosis (RO) water with no added ethanol or with 20, 80, and 92.7% (v/v) ethanol. Under physiological conditions and in conventional experimental setups, such solutions contain salts. As a control sample, the effect of salt on the secondary structure was studied in a solution of RO water with 159 mmol/L NaCl salt. The electrostatic screening by the salt allows assessing the effect of the interactions between the charged terminal units on the secondary structure of the silk protein. The baseline was measured separately for each solvent. The spectra of protein samples were measured immediately after mixing protein and ethanol-containing solvent to prevent the full aggregation of samples in high ethanol concentrations.

Protein secondary structure elements were evaluated from CD spectra using the BeStSel program,^65,66^ single spectrum analysis, disordered–ordered classification, and the secondary structure from PDB-tools. As the secondary structure of CBM is dominantly composed of beta sheets stabilized by hydrogen bonds and ethanol is known to promote the formation of beta sheets and, more generally, intraprotein hydrogen bonds,^23,51,67^ we assume that the secondary structure of the globular terminal units (CBMs) does not experience significant changes with the addition of ethanol. We calculated the change of the secondary structure of the disordered middle part of the protein by subtraction of the CBM contribution of the protein secondary structure from the spectra. The CBM contribution to the spectra and protein secondary structures was calculated using the PDB structure1NBCas the secondary structure from the PDB-tool in BeStSel and subtracting it from the total analysis results based on the sizes of the blocks in CBM-AQ12-CBM.

Ethanol-induced protein aggregation was further characterized by sample imaging (photographs), dynamic light scattering (DLS), and light microscopy. Similar protein solutions were prepared with varied ethanol concentrations (0, 20, 80, and 92.7% (v/v), ethanol, and water filtered) with protein concentrations 0.26 and 0.43 mg/mL, measured by UV/vis-spectrometer at 280 nm. A size measurement was performed with a Malvern Zetasizer Nano. For each sample, three replicate runs each consisting of 10–13 individual scans determined by machine automatic sample quality evaluation were performed. A total of 120 μL of sample solutions was measured using UV-cuvette micro (Brand, 12.5 × 12.5 × 45 mm) directly after sample preparation. The resulting intensity distribution was evaluated. As a comparison volume and number distributions, the values derived from the original intensity distribution were also inspected.

Samples with the same concentrations as in DLS were prepared for microscopy. Two μL of protein solution was pipetted on a glass slide, and a cover glass was applied to prevent sample evaporation and drying. Samples were imaged with a Zeiss Axio Vert A1 microscope using 40× magnification.

### Molecular Dynamics Simulations

**Figure 1:**
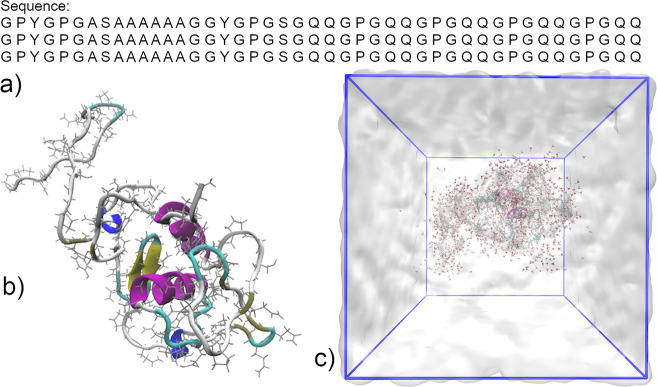
(a) Amino acid sequence of the simulated AQ3 spidroin molecule. (b) Initial structure of the AQ3 spidroin molecule. Color represents the local secondary structure: coil, white; β-sheet, yellow; turn, cyan; β-bridge, tan; 3.10 helix, blue; α-helix, purple. Gray stick visualization indicates the backbone and the amino acid level atomistic structure of the protein. (c) Initial structure for the simulation of the 94% ethanol system. The gray transparent bulk indicates ethanol, water molecules are shown as molecular level visualizations with white and red colors, and the protein molecule corresponds to the configuration shown in (b).

Based on the assumption of ethanol having a minor effect on the CBM and the main effect of alcohol on the protein arising from changes in the intrinsically disordered middle part of the protein, the atomistic detailed MD simulations focused as a model for a spidroin on the AQ3 sequence (see Figure 1a). The sequence consists of three subunits of the intrinsically disordered part of the AQ12 protein examined in the experiments. Notably, the full AQ12 contains 12 hydrophilic–hydrophobic repeat regions.^64,68−71^ An example visualization of the protein in the simulations is shown in Figure 1b. Interactions between atoms in the protein are described by the Amber03ws force field.^72^ This force field was developed for simulations of intrinsically disordered proteins, and it has a good quantitative agreement with experimental structural data.^73^ Our previous studies have also shown its efficiency for the simulation of related proteins.^70,74^ One crucial detail in the simulation of intrinsically disordered proteins is an accurate estimation of interactions with the solvent.^75,76^ To match with the Amber03ws force field, the TIP4P-2005s water model^72,77^ was chosen. The ethanol model was created using AnteChamber Python Parser Interface.^78^ The partial charges on the ethanol molecule were calculated by the R.E.D. server.^79−82^ Quantum mechanical geometry optimization of the molecular configuration was performed by the Hartree–Fock method using the 6-31G(d,p) basis set. The values of partial charges were evaluated by the RESP method.^82^

Simulations were performed by the Gromacs 2022.3 software.^83,84^ To maintain a constant temperature (300 K) and pressure (1 bar) in the simulation system, the Nosé–Hoover thermostat^85,86^ and the Parrinello–Rahman isotropic barostat^87^ were used, with time constants 1 and 3 ps, respectively. Electrostatic interactions were treated using the particle-mesh Ewald (PME) method^88,89^ with a cutoff distance of 1.1 nm. The cutoff distance for Lennard–Jones interaction was 1 nm. To constrain the bond lengths of the hydrogen atoms, the P-LINCS algorithm^90^ was used. The time step was 2 fs. The effect of ethanol on spidroin was studied by simulating each system for 1 μs. The systems with identical amounts of ethanol and water solvent molecules and without protein were simulated for 500 ns to study the effect of the protein on the microstructure of the ethanol/water mixtures.

The protocol for creating the initial configurations and preparing the simulation boxes is provided in the SI. Overall, four different concentrations of ethanol were examined in the simulations. Table 1 summarizes detailed information about the simulation compositions at the molecular level and the sizes of the simulation boxes. The simulation corresponding to pure water contained an additional 0.149 M NaCl salt to match the simulation condition with the experimental ones. Since the AQ3 sequence does not include charged amino acids, a simple monovalent salt in the solvent should not affect the secondary structure of the spidroin in the simulations. This was checked by comparing the response of the spidroin model protein in the solvent with and without salt. No salt was added to the systems with ethanol.

**Table 1:** Summary of the Simulated Systems^a^

vol concn of ethanol (v/v), %	No. of water molecules	No. of ethanol molecules	No. of salt ion pairs (NaCl)	simulation box volume, nm^3^
0	24500	0	71	9.1 × 9.1 × 9.1
20	18209	1408	0	8.8 × 8.8 × 8.8
80	4445	5496	0	8.8 × 8.8 × 8.8
94	1301	6567	0	8.8 × 8.8 × 8.8

a The table presents the solvent composition and the size of the simulation box (final and equilibrated dimensions).

The initial size of the cubic simulation box (9 × 9 × 9 nm^3^) was chosen to prevent the protein from interacting with itself under the periodic simulation box boundary conditions. Notably, the chosen simulation box size is consistent with the empirical box size assumption for modeling intrinsically disordered proteins made by Shabane et al.^75^ They showed that the dimensions of the simulation box where the disordered protein is solvated should be at least four times as large as the radius of gyration of the protein.^75^ The radius of gyration of the AQ3 protein is for the pure water system 1.7 nm ±0.3. The final protein concentration in the simulation box, resulting from one single protein in the system, was ∼26 g/L, and Table 1 summarizes the final box dimensions after the equilibration run (300 ns simulations). Volume concentrations were calculated based on volumes of the pre-equilibrated simulation boxes with the pure component of the mixture. An example of the initial structure of the simulation box is shown in Figure 1c.

For examining the effect of ethanol on the spidroin, the first 300 ns of the 1 μs simulation were considered as equilibration time and disregarded in data analysis. The remaining 700 ns were treated as the production run, and the main analyses correspond to this time period. The equilibration duration was determined based on the time dependencies of the structural characteristics in the investigated system. Root-mean-square deviation of the protein structure (all heavy atoms) was used to determine the equilibration time of the spidroin structure. Solvent structure and solvent environment equilibration time were determined based on time evolution in intermolecular hydrogen bonds of the protein vs intramolecular hydrogen bonds. A further equilibration measure for the solvent structure was the evolution of the time average size of the water and ethanol clusters in the system. These dependencies are shown in Supporting Information, Figure S1.

The analysis was performed using the Gromacs tools^83,84^ and personal scripts. Distributions are presented in this study as probability density functions and plotted using kernel density estimation. Errors were calculated as standard deviations.

## Results

### Experimental Results on Ethanol-Induced Secondary Structure Changes: Circular Dichroism

**Figure 2:**
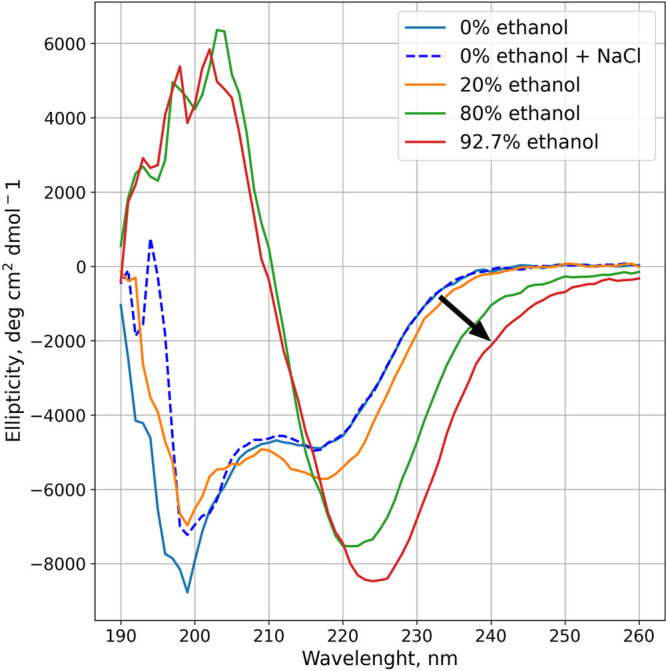
Experimental CD spectra characterizing the secondary structure of CBM-AQ12-CBM in different solvents. The blue line represents the protein in water, and the dashed blue line represents the protein in water with the presence of salt (NaCl); orange, green, and red lines represent protein in ethanol–water mixtures containing 20, 80, and 92.7% ethanol (v/v), respectively. Related high-tension voltage graphs are shown in Figure S2. Arrow illustrates the shift of the spectra with an increase in the ethanol concentration.

The circular dichroism spectra corresponding to CBM-AQ12-CBM solutions with varying amounts of ethanol added to the solvent phase are presented in Figure 2. The data show that in water, the spectra of the protein have two distinctive negative peaks, one around 198 nm and the other around 216 nm. These indicate a random coil structure and β-sheet structure, respectively.^91^ It is important to remember that the spectra of Figure 2 include the contributions of both the spidroin part (AQ12) and the terminal domains (CBMs); distinguishing the two parts might not be straightforward. In literature, the spidroin is reported to have a mostly random coil structure in water.^37,92−94^ The CBM, on the other hand, is highly organized into β-sheets.^63,95^ This indicates good agreement between the experimental results and data reported in the literature. Notably, the addition of 159 mmol NaCl does not change the secondary structure of CBM-AQ12-CBM. However, below 200 nm, some differences are visible, but in this region, the added NaCl strongly affects the measurement.^96^ Notably, the high-tension voltage is increased to a value above 1000 V (Figure S2), where it hits the measurement ceiling, which makes the measurement unreliable. Since in the wavelength range between 200 and 260 nm the spectra corresponding to the added salt system do not show any difference to those measured for the protein in water without the added salt and the trend is also similar at wavelengths below 200 nm, it is reasonable to conclude that the NaCl does not change the secondary structure of CBM-AQ12-CBM. The increase in noise of all spectra below 200 nm results from a moderate increase in the measured high-tension voltage values. Therefore, the exact number values corresponding to the spectra in that region are not so reliable for any of the spectra. However, the trends can be inspected.

Adding 20% (v/v) ethanol induces changes in the secondary structure. The negative peak at 198 nm decreases in magnitude, indicating less random coil content. An increase of the negative peak around 216 nm and a shift of the peak maximum to 217–218 nm point to an increase in ordered structures. The addition of 80% ethanol induces more significant changes in the secondary structure. The negative peak at 198 nm vanishes completely, and the spectrum becomes strongly positive. The peak at 218 nm increases in magnitude and shifts to a higher wavelength (222 nm). In the 92.7% ethanol solution, the negative peak strengthens further in magnitude and shifts to 225 nm. The spectra shape of the protein in 80% and 92.7% ethanol solutions matches the shape rising from β-sheets in the sample, with a positive peak at 198 nm and a single negative peak above 210 nm.^91^ However, simply based on the CD spectra, distinguishing ordered structures, α-helices, and β-sheets from each other is challenging due to overlap of the characteristic signature peaks. For α-helices, a single positive peak at 192 nm and negative peaks at 208 and 222 nm would be expected.^91^ Our spectra do not show signs of two negative peaks. However, the negative peak in 92.7% ethanol solution is closer to 222 nm (α-helices) than to 216 nm (β-sheets). At the elevated ethanol concentration, the signal of the negative peak is almost as strong as the original peak for the random coil, which could indicate the possible presence of α-helices.^91^

**Figure 3:**
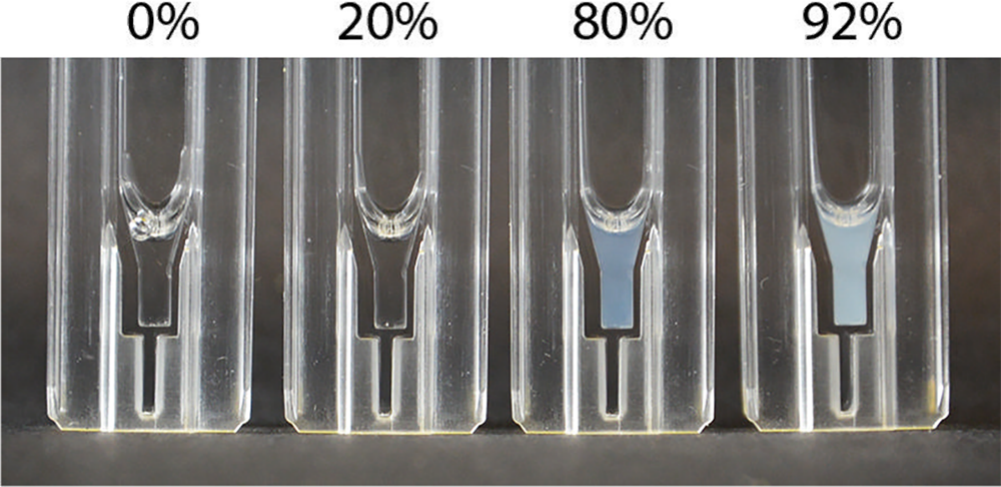
Photograph of protein samples in different solvent compositions. The percentage refers to the ethanol content in the volume percentage. In 80 and 92% ethanol solutions, the samples have a visibly turbid appearance, with the turbidity increasing with the ethanol content.

Overall, the peak shift to a higher wavelength (to right) indicates an increase in order.^37^ Here, the observed shift could be an indication of a more ordered structure, not just of a single protein molecule secondary structure, but also due to some extent of aggregation. Alcohol (ethanol and methanol) is known to precipitate silk protein in high concentrations.^37,97−104^ Consistent with this, indeed, in our samples at high ethanol concentrations, aggregation was visible (Figure 3). In the 80% ethanol solution, the protein sample turned slightly turbid cloudy, indicating the presence of microaggregates. However, the aggregates remained as a colloidal suspension, and it was not possible to separate and sediment them with centrifugation. In the 92.7% ethanol solution, the protein sample also turned turbid cloudy, but some larger aggregates started to form very quickly. As mentioned, the CD measurement was done immediately after mixing the protein with an ethanol solution. This was to prevent the aggregation from progressing too far before measurement. In 1 h, the protein sample in the 92.7% ethanol solution was completely aggregated (Figure S3). This led to no soluble protein left in the solution, resulting in the absence of a signal in the CD spectrum.

The results of DLS measurements (Figure S4) and light microscopy images (Figure S5) show that the particle size does increase with increased ethanol concentration. Even a 20% ethanol solution facilitates the formation of protein nanoclusters. As the ethanol concentration increases, both the size and the number of clusters grow significantly (Table S1). Numerical results of DLS analysis (mean size based on intensity, mean size based on the number of particles, polydispersity index, and count rate) are presented in Table S1. DLS is unable to differentiate the aggregate size in the most elevated ethanol concentrations, and the results show the same average size of nanoclusters at higher protein concentrations. However, increased ethanol content results in an increase in the number of clusters and acceleration of the aggregation over time in the DLS data. Consistent with this, light microscopy also shows the appearance and growth of microclusters in samples with 80 and 92.7% ethanol, but it is also able to detect directly that the added ethanol increases the speed of aggregation, leading finally to full aggregation (Figure S5). The observed formation of nanoclusters in significant amounts and the increase in microaggregate size explain the emergence of the cloudy turbidity and its increase with ethanol in the samples shown in Figure 3.

In conclusion, the CD spectra indicate that the ethanol changes the silk secondary structure from a disordered random coil-dominated structure to an ordered β-sheet-rich state. The observed trend and interpretation match the observations of Dicko et al. on the secondary structure of the spidroin in solution and the effects of methanol on it.^37^ Dicko et al. observed a similar gradual spectral change from measurement data presenting a strong random coil peak to data with a strong β-sheet peak along with a shift of the peaks to higher wavelengths. The latter indicates an increase in order and final irreversible precipitation of silk after a few hours. The similar observations of Dicko et al.^37^ to the findings in this current work validate our results and interpretation of the spectra.

Numerical percentage estimates of secondary structure fractions were calculated with BeStSel to enable a comparison of the experimental results with the simulations. The results based on disordered–ordered classification were clear and in accordance with the literature. In water, the silk is predominantly disordered, but the addition of ethanol, especially at elevated concentrations, induces change into a dominantly ordered secondary structure. Table 2 collects the calculated percentage fractions of different secondary structure features. Addition of ethanol results in random-coil content decreasing, as well as clear changes in the spectra. Coil content, on the other hand, is insensitive to ethanol content between 20 and 80 or 92.7% ethanol. This is surprising since the change in the random coil region of the spectra corresponding to solvent compositions between 20 and 80% ethanol concentration is so significant. Ethanol increases β-sheet content, and the effect is more pronounced with larger ethanol concentration. At ethanol concentrations between 80 and 92.7%, the analysis results suggest a slight decrease in the β-sheet content. This could result from the small peak shift from 222 to 225 nm. The longer wavelength is farther from the ideal β-sheet peak wavelength. As mentioned earlier, spidroin aggregation occurs time-dependently, with the higher ethanol concentration precipitating the protein faster. The difference in peak position between the 80 and 92.7% ethanol solutions could result from different aggregation stages in the system. Alcohol has, in prior silk fiber studies, been reported to change the fibers into water-insoluble and dominantly β-sheet containing structures.^97,99−101^ This suggests that the β-sheet content either stays constant or even increases during aggregation; however, a decrease is not expected.

**Table 2:** Single Spectra Analysis of Experimental CD Spectra with BeStSel to Extract the Secondary Structure Fraction^a^

ethanol concn (v/v), %	coil	β-sheet	turn	helix
0	0.50	0.36	0.12	0.02
20	0.40	0.45	0.14	0.02
80	0.39	0.50	0.12	0.00
92.7	0.40	0.47	0.12	0.00

a The analysis is performed for the wavelength region of 190–250 nm.

Ethanol does not change the turn content of the sample. For helices, the analysis suggests a very low content in all samples and a complete disappearance of the helix content with an elevated ethanol concentration in the solvent. This low helix content for the silk protein in water is an underestimation: notably, silk in water solution and water-soluble silk fibers are widely reported to have α-helices as a major component of their secondary structure.^64,97,99−101,105,106^ As mentioned earlier, the overlap of α-helix and β-sheet peaks in the CD spectra makes it challenging to distinguish them in the data, particularly in systems that can be expected to contain both α-helices and β-sheets. This difficulty can lead to inaccurate analysis interpretations of the data, especially in terms of percentage fractions of secondary structure components in a complex system such as ours. This may explain the low content of helices.^107^ Prior to us, helical content decreasing due to ethanol treatment has been reported in the literature, but usually, this is stated as a change from helices to β-sheets as the dominant structure.^97,100,101,105^ Here the analysis suggests a complete disappearance. Opposed to helix content decreasing, some studies report the helical content to stay relatively constant and the fraction of helical structure significant.^99,104^ Because of these reasons, we believe that the actual helix content is overall higher than that predicted by the performed analysis; the complete disappearance of helix content in this silk protein system is unrealistic. However, a decreasing trend in the helix content with ethanol addition is possible.

Overall, the trends observed with the secondary structure changes in the BeStSel analysis with ethanol solvent additive (see Table 2 for summary) follow our direct interpretation of the measured spectra shapes and peak positions and are consistent with the literature.^100,106^ Ethanol as a solvent additive decreases the random coil content and converts it to mainly β-sheets. Additionally, the helix content is decreased. In prior literature, very sparse numerical data for secondary structure fraction changes based on experimental studies of the alcohol effect on silk proteins exist because of the low accuracy of CD spectra-based secondary structure content analysis for proteins with multiple different forms of secondary structures. Moreover, a quantitative comparison with the literature data is challenging due to the difference in the samples and experimental setups.

### Simulations vs Experimental Characterization Results on a Silk Protein Secondary Structure in Water

**Figure 4:**
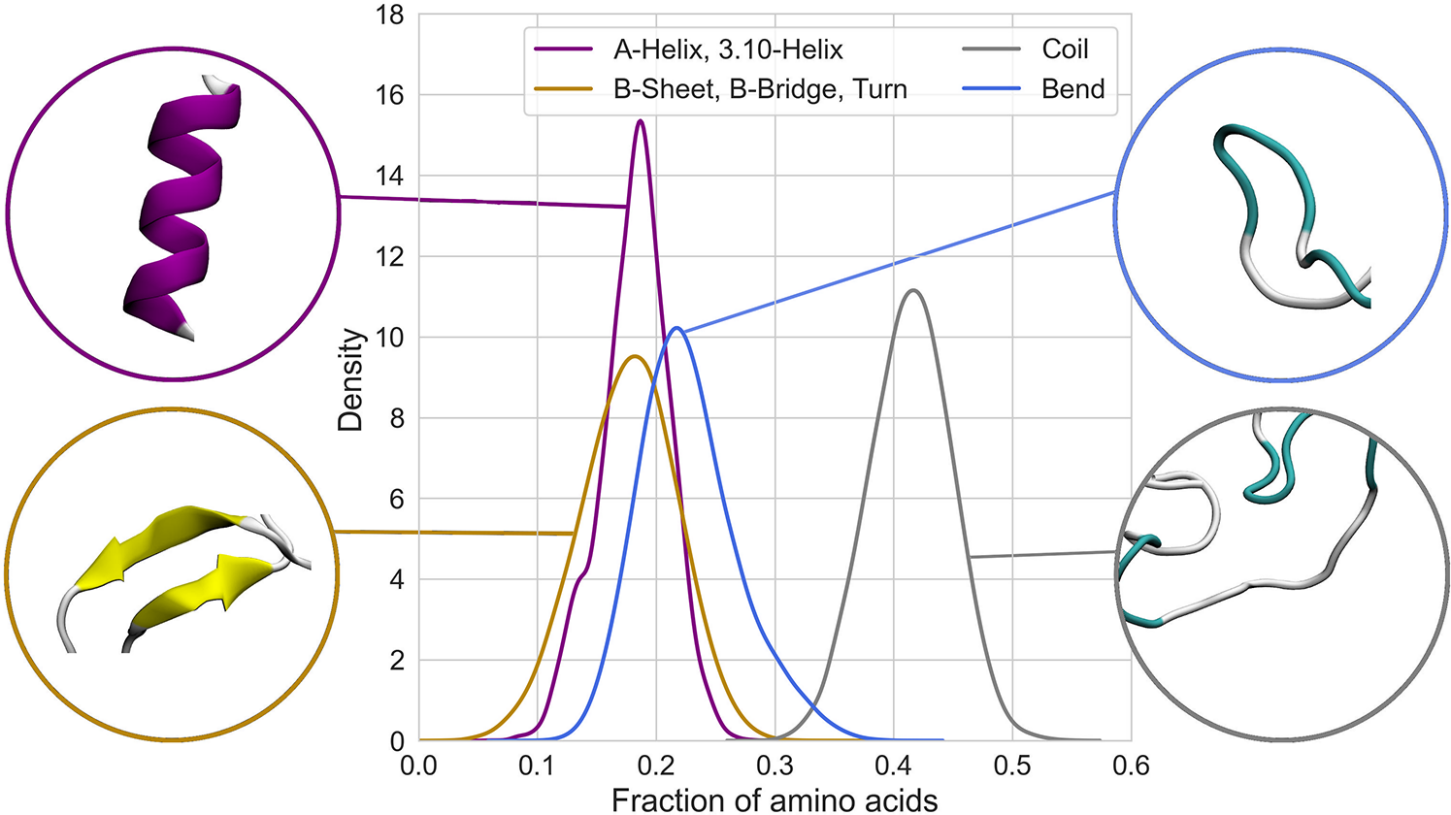
Probability density distribution corresponding to secondary structure fractions for spidroin in water. The distribution shows a summary of the secondary structure. Detailed distribution is shown in Figure S6 in the Supporting Information.

To resolve the effects of ethanol as a solvent additive, namely, the ethanol-induced aggregation of silk protein molecules and the effect of ethanol on the secondary structure of the individual protein molecules, we resort to MD simulations. In the simulations, a shorter AQ3 intrinsically disordered repeat sequence (Figure 1) than the middle part of the experimentally studied CBM-AQ12-CBM protein was examined. The AQ3 secondary structure in the MD simulations was estimated using the standard algorithm DSSP.^108,109^Figure 4 shows the resulting time-averaged distributions of secondary structures grouping helical (α- and 3.10-helices), turn-related (turns, β-sheets, and β-bridges), coil, and bend structures as distributions. Figure S6 presents the corresponding detailed distributions. The diversity of secondary structures observed in the simulations is consistent with recent comprehensive experimental studies of the secondary structure of spider silk proteins.^106,110,111^ Based on the prior studies, alanine-rich hydrophobic regions in individual silk protein molecules tend to form α-helices. In the simulation, these regions also form short-living 3.10-helices. In contrast, the glycine-rich region tends to be in a random coil state. It can also form bends and turns, stabilized by intramolecular hydrogen bonds that support the formation of β-bridges or small β-sheet structures.

For validation of the simulation results, we resort to comparing the total ordered structures (helices and β-sheets), turns, and other structures (bend and coil) in the simulations with the CD characterization results. This is because making a detailed distinction between ordered structures (α-, 3.10-helices, and β-sheets) in the CD data shown in Figure 2 is challenging. The results and a comparison of the fraction of ordered structure in the intrinsically disordered part of the protein (AQ3 in simulations and AQ12 in experiments) are presented in Table 3. Notably, as the experimental characterization results (Table 2) correspond to CBM-AQ12-CBM, the data presented in Table 3 for the experiments have been calculated by subtracting from the secondary structure the estimated contributions of the linker segments connecting the CBM terminal units and the middle part and the contributions rising from CBM terminal units. Notably, the linker segments are small, corresponding to less than 5% of the protein and mainly helical in structure, and their contribution was estimated based on our previous simulation study of the CBM-AQ3-CBM protein.^70^ The CBM units are rich in β-sheets and secondary structure composition calculated based on the PDB structure of 1NBC using the PDB tool in BeStSel.

**Table 3:** Comparison of the Secondary Structure Content of the AQ12 Spidroin in Water in the CD Experiments (Intrinsically Disordered Region Only, Linker, and CBM Terminal Unit Contributions Subtracted) and AQ3 in the MD Simulations^a^

	ordered	turns	others
experiments (CD)	0.28	0.14	0.58
simulations (MD)	0.21 ± 0.03	0.15 ± 0.03	0.64 ± 0.04

a To enable a comparison, the secondary structures are combined as ordered (α-, 3.10-helices, and β-sheets), turns, and other (bend and coil).

Data of Table 3 show that the experimental and simulation results are in good agreement. This supports good accuracy of the force field, but also the absence of the additional supramolecular structures formed via aggregation in the experiments. The ordering of the silk protein should occur mainly in the alanine-rich regions in the AQ12 protein, contributing ∼0.18 of the disordered middle part. As both the experiments and simulations estimated ordered structure content is higher than 0.18, ordered structures are present also in the glycine-rich regions. In the simulations, helical structures contribute 0.18 ± 0.03 (see Figure 4), with the rest of the estimated 0.21 ± 0.03 ordered structure rising from turns fixed by small β-sheet structures in the glycine-rich regions. Here, the spidroin protein is free and is dilute in solution. Opposed to this, in fibrils, in the glycine-rich region the fraction of turns should be less, and instead, a larger fraction of β-sheets can be expected due to the stretching of molecules parallel to each other. The fraction of the secondary structures (α- and 3.10-helices, β-sheets, turns, and bridges) observed in our simulation (0.36 ± 0.05) is close to the value for the spidroin in fibril obtained by Wang et al. (∼0.44).^106^ However, a detailed quantitative comparison with literature data on the secondary structure of the silk protein in fibrils is difficult due to the large variation in the supramolecular structures and deviations in the amino acid sequences.

### Simulations vs Experimental Characterization Results on the Silk Protein Secondary Structure in Water/Ethanol Mixtures

**Figure 5:**
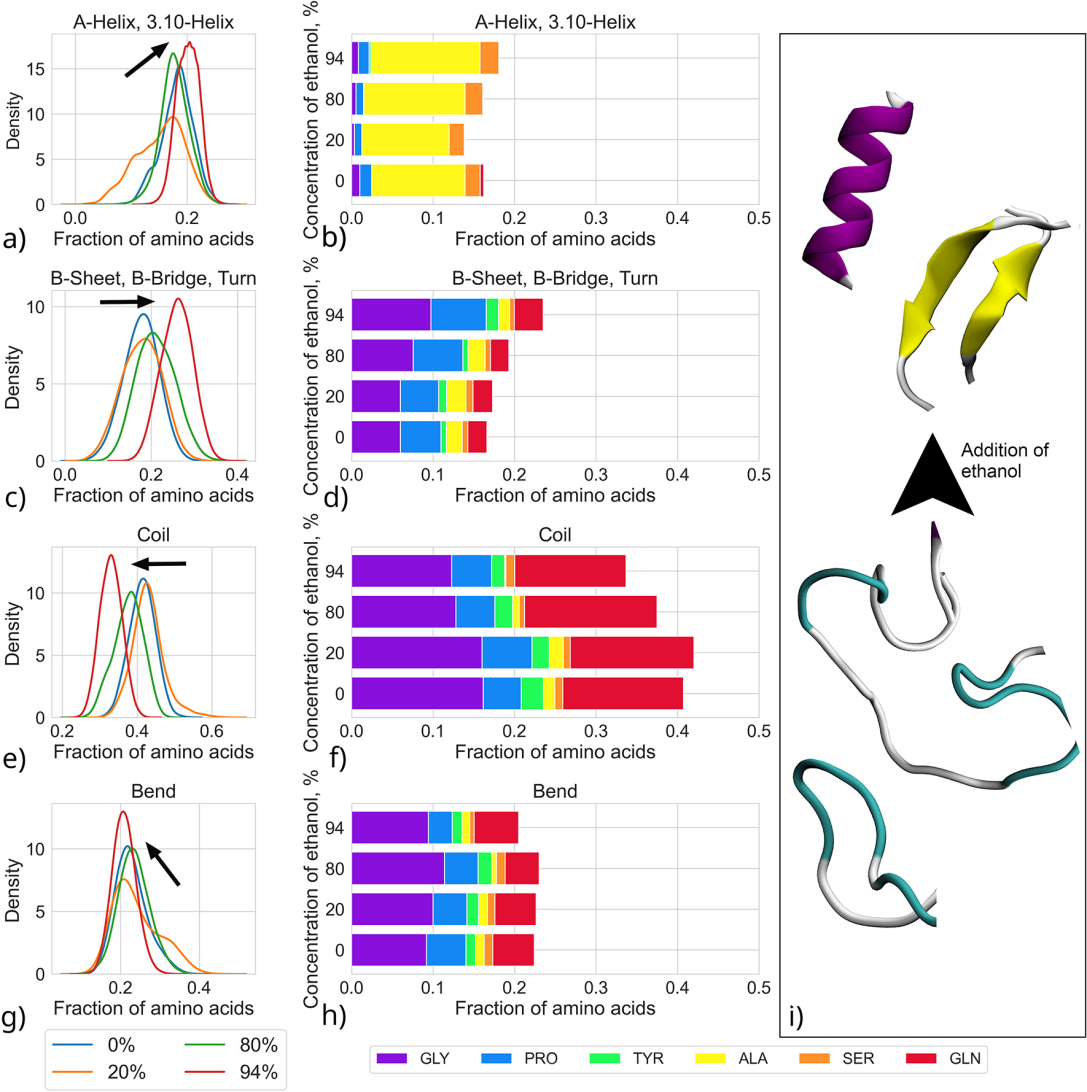
Probability density distributions of different secondary structures and amino acid contributions to each probability distribution in the water/ethanol systems with varying ethanol content in the MD simulations. (a) and (b) present α- and 3.10-helices, (c) and (d) present turns fixed by β-sheets and bridges, (e) and (f) present coils, and (g) and (h) present bend contributions. The arrows point the shift in the distributions with an ethanol concentration increase. A fraction of 1.0 refers to all amino acids in the AQ3 molecule. Panel (i) illustrates schematically the transition in secondary structure with the addition of ethanol. Figure S7 in the Supporting Information shows the original distributions based on which the averages are calculated.

Consistent with the CD characterization, the MD simulations also show an increase in the order of the spidroin structure with the addition of ethanol. Figure 5 illustrates the changes in grouped secondary structure distributions with the addition of ethanol into the system (panels a, c, e, and g) and the contributions of specific amino acid species to these distributions (panels b, d, f, h). Secondary structure fractions calculated as averages based on these distributions are present in Table S2. Figure S7 shows the original distributions behind the averages presented in Figure 5.

The most pronounced changes by ethanol occur in the simulations in the fraction of the coil structure. In the pure water vs 94% ethanol solvents, the fraction of the disordered state of the protein (bends and coil) differs by 0.10 ± 0.04. The change fraction is smaller than the changes in the secondary structure in our CD experiments (see Table 2). This can be related to additional ordering due to the aggregation taking place. However, different authors^30,112^ have reported that, in silk fibers in which the aggregation state of the protein does not change with the addition of alcohol, alcohol as a solvent additive leads to a decrease in disordered content (∼0.10).

In the simulations, the changes in the coil fraction are associated with an increase in the turn content. The changes in α-helix fraction remain less pronounced, with the α-helix fraction remaining constant within the error estimate when ethanol is introduced. Namely, the difference between α-helix fraction in pure water and 94% ethanol solvent is 0.02 ± 0.04. Protein secondary structure distribution peaks become narrower with addition of ethanol, being narrowest in the 94% ethanol solvent. This could indicate that ethanol addition reduces the number of possible conformation states for the protein molecule.

It is also important to note that in pure water and the 20% ethanol solvent systems one of the α-helices transitioned temporarily into the bend/coil state. This affected the final secondary structure fraction distributions for these systems. The event occurred only once during the simulation duration of 1 μs for both systems and was not observed at higher ethanol concentrations. Such is expected due to α-helix stabilization by ethanol addition; see below. Altogether, the increase in the ordered structure taking place with increasing ethanol content of the solvent in the simulations (Figure 5) is less pronounced than in the experiments (Figure 2). This is related to ethanol also provoking aggregation of silk proteins in the experimental setup and the formation of resulting supramolecular structures affecting the CD results. Simulation, in turn, shows the effect of ethanol on an infinitely dilute solution of the silk protein. Table 4 shows a comparison of the CD measurements and simulation-derived structural changes due to ethanol in the solvent.

**Table 4:** Comparison of the Spidroin Secondary Structure Content Derived from the CD Experiments and MD Simulations^a^

	ordered		turns		others	
ethanol concn (v/v), %	exp.	sim.	exp.	sim.	exp.	sim.
0	0.28	0.21 ± 0.03	0.14	0.15 ± 0.03	0.58	0.64 ± 0.04
20	0.43	0.18 ± 0.05	0.16	0.15 ± 0.04	0.41	0.67 ± 0.07
80	0.48	0.21 ± 0.03	0.13	0.18 ± 0.03	0.39	0.61 ± 0.04
92.7 (94)	0.44	0.27 ± 0.03	0.14	0.19 ± 0.03	0.41	0.54 ± 0.03

a The highest ethanol concentrations for the simulation and experiment systems are different (94% and 92.7%, respectively). The secondary structures are grouped as ordered (α-, 3.10-helices, and β-sheets), turns, and other structures (bend and coil). Values show the fraction of amino acids involved in specific secondary structures.

The simulations also allow pinpointing the changes at individual amino acid level. Figure 5b, d, f, and h show clearly that alcohol as a solvent additive affects the disordered part of the spidroin more than the ordered alanine-rich regions. The number of turns, fixed by β-sheets and bridges in the structure, increases. A further summary of the amino acid specific contributions is provided in Table 5, which shows the difference in the secondary structures between pure water and 94% ethanol systems for each amino acid type.

**Table 5:** Amino Acid Type Specific Differences in the Spidroin Secondary Structure Changes between Pure Water and 94% Ethanol Solvent Systems for Each Amino Acid Type in the AQ3 Sequence in the MD Simulations^a^

	GLY, ×10^–2^	PRO, ×10^–2^	ALA, ×10^–2^	GLN, ×10^–2^	TYR, ×10^–2^	SER, ×10^–2^
helix	–0.16 ± 1.24	–0.15 ± 0.89	1.92 ± 2.36	–0.37 ± 0.65	0.23 ± 0.40	0.47 ± 0.59
turn	3.73 ± 2.89	1.82 ± 1.94	–0.62 ± 1.81	1.19 ± 1.94	0.87 ± 0.64	–0.11 ± 0.83
coil	–3.87 ± 2.91	0.25 ± 1.46	–1.26 ± 1.35	–1.18 ± 3.28	–1.13 ± 0.97	0.12 ± 0.96
bend	0.23 ± 2.98	–1.92 ± 2.26	–0.13 ± 1.06	0.35 ± 2.90	0.03 ± 0.92	–0.48 ± 0.82

a Values show the difference in the fraction of AQ3 amino acids involved in specific secondary structures between the two solvents. A positive value indicates an increase in the fraction with the addition of ethanol, and a negative one indicates a decrease. Amino acids are arranged in the table in decreasing order from the largest total ethanol influence (GLY) to the smallest one (SER).

At the amino acid level, most affected by ethanol are the glycine residues of AQ3. Glycine has the largest degree of conformational flexibility compared to the other amino acids. With ethanol addition, amino acids in the glycine-rich region change from random coil and bend-related conformations to those corresponding to turns. The alanine-rich region experiences an increase in the number of helical conformations. In pure water and low ethanol concentration solvents, alanine and serine that are amino acids in the alanine-rich region of the silk protein form also other structures besides α-helices. However, an increase in the ethanol concentration decreases the total amount of these additional structures, consistent with ethanol stabilizing α-helices. The results also suggest that in fibers, in which the alanine-rich regions form β-sheets, the addition of ethanol will stabilize existing β-sheets and slightly enlarge the β-sheet crystals. This structural change occurs via the disordered glycine-rich regions, partially contributing to these structural elements.

### Distribution of H-bonds in the silk protein/water/ethanol mixtures

**Figure 6:**
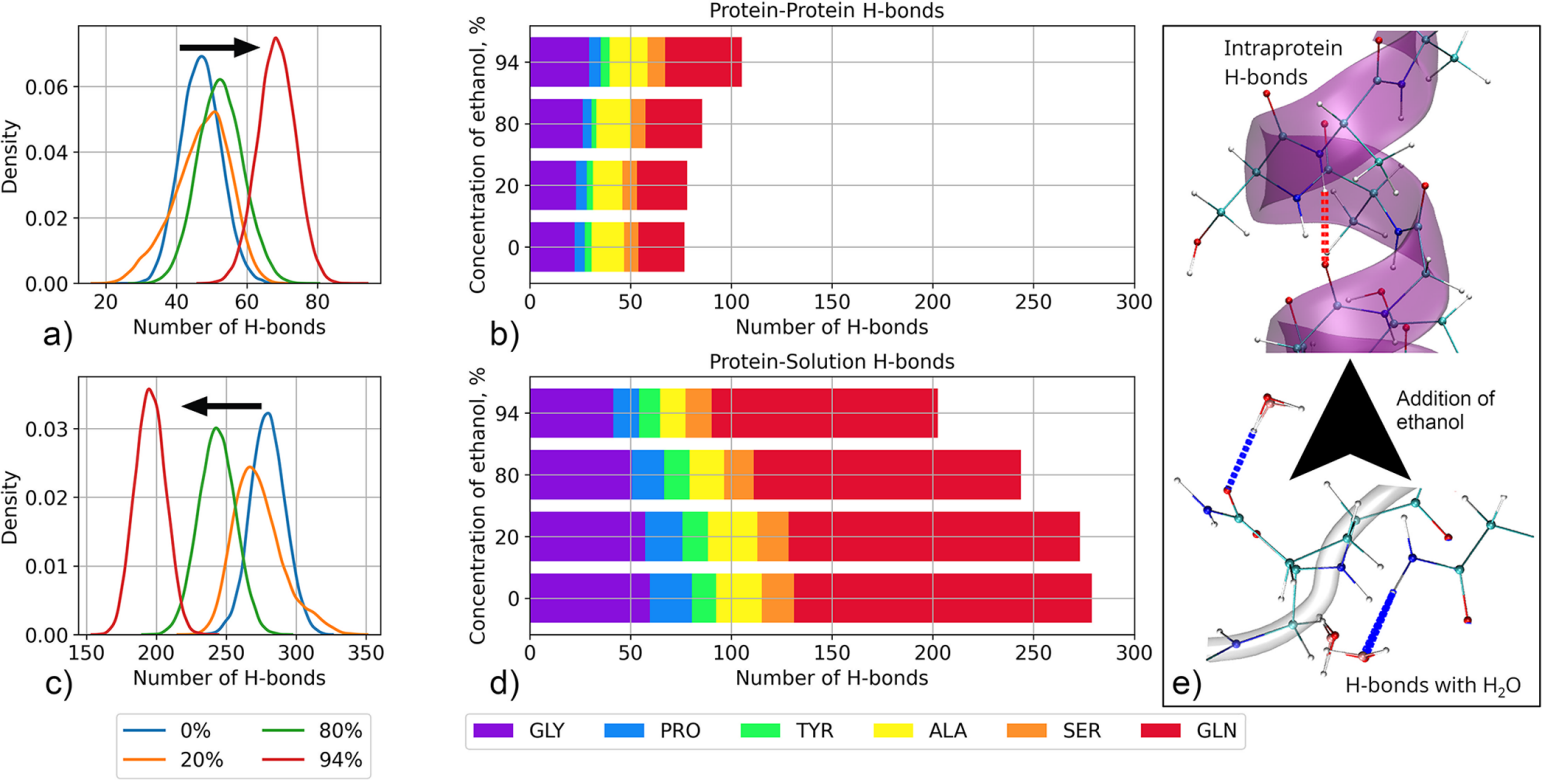
Probability density distribution of (a) intramolecular protein hydrogen bonds and (c) intermolecular hydrogen bonds between the protein and solvent molecules. The arrows point to the shift of the distributions with an increase in ethanol concentration increase. (b, d) Averaged contributions of the different amino acids for intramolecular protein hydrogen bonds and interprotein hydrogen bonds, respectively, in the different solvent compositions. (e) Illustrates the transition in hydrogen bonding with the addition of ethanol. Figure S8 in the Supporting Information shows the original distributions based on which the averages are calculated.

Conformational changes in a protein, such as secondary structure changes, are associated with a difference in the balance of intramolecular and intermolecular interactions. To examine this, we analyzed the hydrogen bonding of the protein, both intramolecular hydrogen bonds and those formed with the solvent. The distribution of intramolecular protein hydrogen bonds and those formed with the solvent molecules, as well as the contributions of the different amino acids to these distributions, is shown in Figure 6. Figure S8 presents the distribution of hydrogen bonds for each amino acid type.

The data of Figure 6 show that adding ethanol reduced the number of hydrogen bonds by the protein with the solvent and increased intramolecular interactions. This is quite expected as the formation of additional turns in the glycine-rich region and stabilization of α-helix in the alanine-rich region requires the formation of additional intramolecular H-bonds. The number of intramolecular hydrogen bonds increased by a factor of ∼1.4 in the 94% ethanol concentration solvent. On the other hand, hydrogen bonding with solvent is reduced by a factor of ∼1.3. Table 6 shows the difference in the number of hydrogen bonds for pure water and 94% ethanol systems for every type of amino acid.

**Table 6:** Change in the Mean Number of Intramolecular Protein Hydrogen Bonds and Protein–Solvent Intermolecular Hydrogen Bonds Per Amino Acid in Solvent Systems of Pure Water and 94% Ethanol Solvent for Each Amino Acid Type in the AQ3 Molecule^a^

	GLN	SER	ALA	GLY	PRO	TYR
intra	0.51 ± 0.23	0.27 ± 0.44	0.14 ± 0.16	0.15 ± 0.13	0.04 ± 0.12	0.13 ± 0.36
inter	–1.18 ± 0.39	–0.51 ± 0.53	–0.48 ± 0.18	–0.38 ± 0.18	–0.38 ± 0.18	–0.26 ± 0.52

a A positive value indicates the number of hydrogen bonds increases and negative points to a decrease in hydrogen bonding. Amino acids are arranged in an order of decreasing hydrogen bond number count change magnitude.

The most significant changes in the balance of inter/intramolecular interactions with the addition of ethanol occur with glutamine. Glutamine is the most polar amino acid in the AQ3 silk protein structure. It has the longest side chain and can form multiple hydrogen bonds (∼5–6). In water solution, most of these hydrogen bonds are with water molecules, i.e., intermolecular. Addition of ethanol replaces part of the compounds with intramolecular hydrogen bonds. The other amino acids of the protein exhibit the same trend in their hydrogen bonding; however, the changes in their interactions are not so significant. In the ordered region of AQ3 spidroin, interactions of serine are most affected by the addition of ethanol. Serine is also a polar amino acid, and it has a hydroxyl group that can form an additional hydrogen bond with the surroundings. The hydrogen bonding changes point to a conclusion that the effect of ethanol depends not only on the polarity of the amino acids in the silk protein sequence, but also on the ability of the amino acid to form hydrogen bonds. Thus, a change in the number of polar amino acids in a protein should change the strength of the effect of the alcohol on the resulting solution and assembly. The observed changes in the hydrogen bonding point to the effect of ethanol depending on the polarity of the amino acids in the silk protein sequence but also indicate that the ability of the amino acid to form hydrogen bonds is important in its response to ethanol. Based on this, we expect that the number of polar amino acids in a protein affects the effect of alcohol on the protein solution and assembly. Notably, our observations here are in agreement with the recent study of the effect of ethanol on the secondary structure of chicken villin headpiece (HP-36) protein.^113^ The study showed the indirect key role of the hydrophilic amino acids in ethanol-induced unfolding.

### Molecular Level Distribution of the Solvent along the Silk Protein Chain

**Figure 7:**
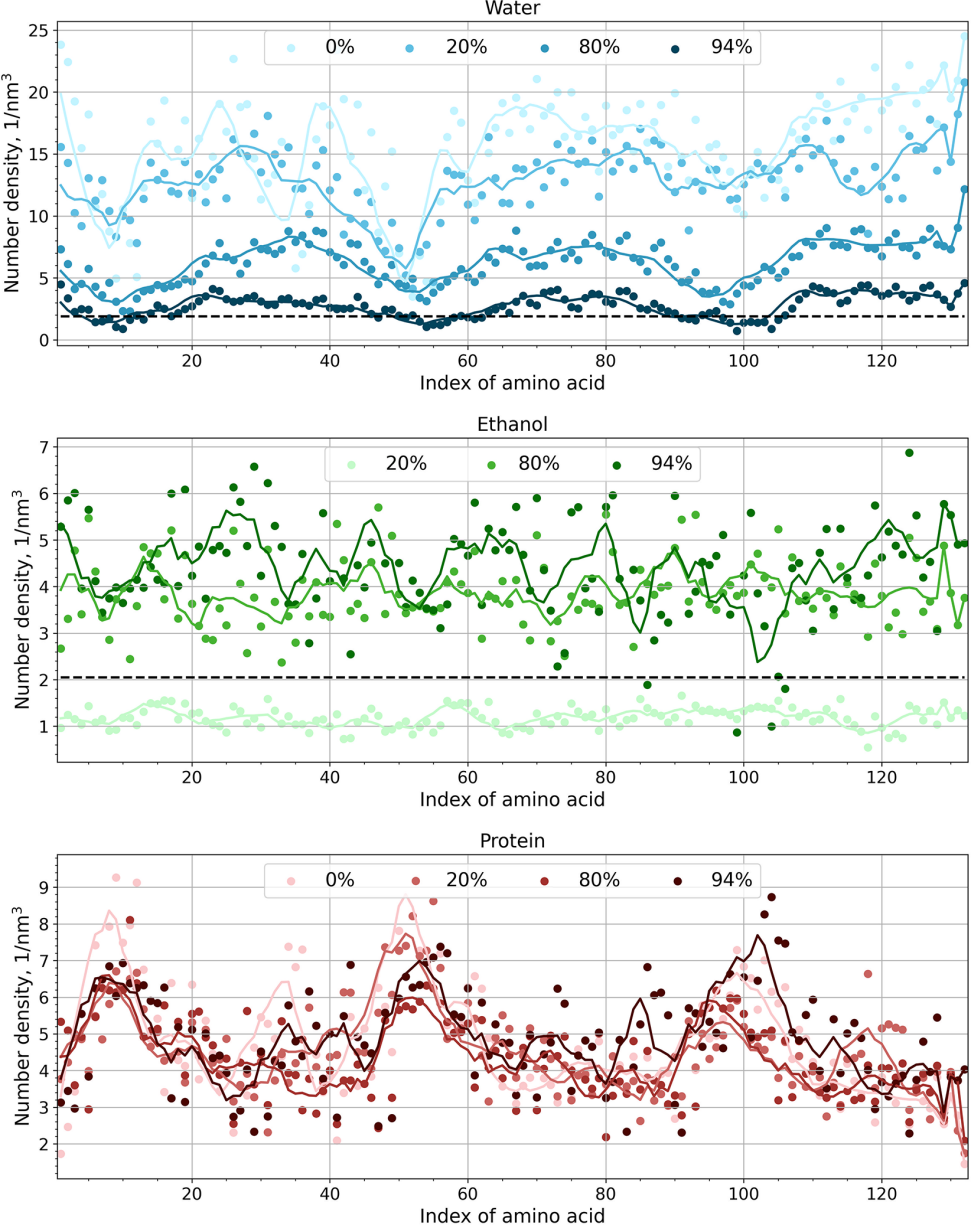
Local number density of the solvent molecules (water and ethanol) and amino acids of the protein around each amino acid in the protein chain. Solid lines show the moving average for each set. The dashed lines indicate the average bulk density of the solvent component in the system of its lowest concentration. For the 94% ethanol system, the dashed line shows water bulk density. For the 20% ethanol system, the ethanol bulk density is indicated. The detailed, unnormalized data corresponding to each amino acid in the protein chain are shown in Figures S12 and S13.

Ethanol/water mixtures are known to be heterogeneous at the microscale.^58−60^ The MD simulations allow for studying how this heterogeneity affects the solvation of the AQ3 silk protein. Figure 7 presents the density of solvent molecules (water and ethanol) around each amino acid along the protein chain. Corresponding unnormalized data with each amino acid distinguished are shown in Figures S12 and S13. The analysis procedure is described in the SI.

To understand the data in Figure 7, it is useful to consider the primary and secondary structures of the protein as the molecular surroundings of the amino acids mainly determined by this. For example, the hydrophobic helical alanine-rich regions (amino acid indices 7–14, 51–58, and 95–102) have fewer solvent molecules and more contact with the neighboring amino acids than the glycine-rich regions. The difference in solvent density around the hydrophilic and hydrophobic regions results mainly from the water distribution. While ethanol molecules can interact with both hydrophobic and hydrophilic regions of the protein and are evenly distributed along the protein chain, water molecules solvate mainly the hydrophilic, glycine-rich regions. This can be seen in clear oscillations in the water molecule data according to the regions.

**Figure 8:**
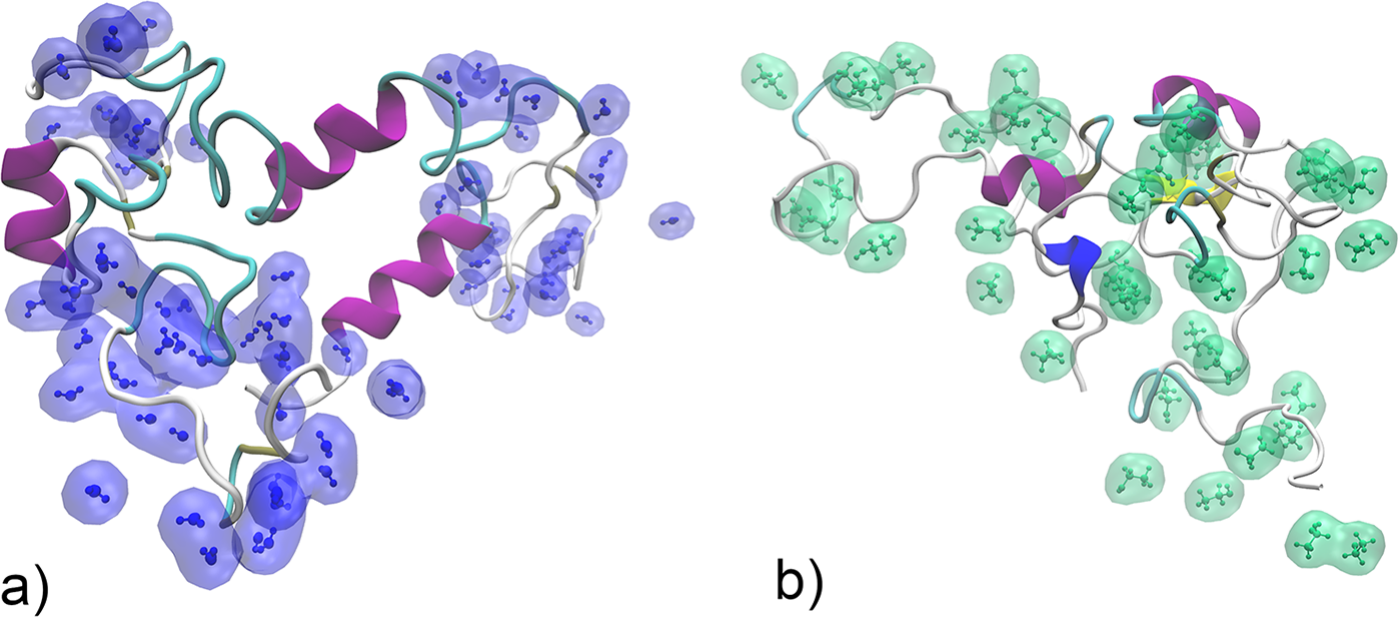
Visualization of the distribution of the solvent around the protein molecule. (a) Distribution of the water molecules (blue color) in the 94% ethanol concentration solvent system. (b) Distribution of ethanol molecules (green color) in the 20% ethanol concentration solvent system. The visualized molecules correspond to those solvent molecules that were located within 0.25 nm cutoff distance from the protein. Distance was measured as the distance of any atom in the solvent molecule to any atom in the protein. Solvent clusters are highlighted in the visualization by transparent shading.

Comparison of the local density of the solvent molecules around the amino acids and the bulk density of the solvent molecules shows that the concentration of water around the hydrophilic disordered regions of the protein is higher than that in the bulk. For details of the local density calculation around each amino acid, see SI. Calculated as the number of solvent molecules divided by the volume of the simulation box, a value of ∼1.89 1/nm^3^ is obtained for the bulk water. The concentration of ethanol around the protein is always lower than the bulk concentration (∼2.05 1/nm^3^). This indicates that ethanol in the solution decreases the level of interaction between the protein and the solvent. The finding correlates with the conclusions of the hydrogen bond analysis discussed above. A typical example of solvent distribution around the protein molecule is presented in the snapshots shown in Figure 8. Figure 8a shows water localization near the protein in the 94% ethanol solvent system, and Figure 8b visualizes the locations of ethanol near the protein chain in the 20% ethanol solvent system.

As shown by both Figure 7 and Figure 8, even at a high ethanol concentration, water molecules hydrate the protein and form clusters around the disordered regions of the protein. The hydrophobic, ordered regions remain unhydrated. In contrast, ethanol solvates both disordered and ordered regions. This is because ethanol molecules can form hydrophobic contacts with the alanine-rich helices and hydrogen bond with amino acids in disordered regions. As a result, ethanol molecules are uniformly distributed along the protein chain as separate molecules that do not form visible molecule clusters, as visible with solvation with water. The difference in water and ethanol molecular level solvation and distribution around the protein is an important result in terms of the effect of ethanol on the interaction of silk protein with the environment. A direct significance is that ethanol also significantly weakens hydrophobic interactions in the system via the solvation of hydrophobic regions. This is evident in the radial distribution functions calculated between alanines located in different alanine-rich regions (Figure S14). Even a small ethanol addition significantly reduces contacts between different α-helices, i.e., folding conformations of the AQ3 change. The role of the middle part helix–helix contacts in the CBM-AQ3-CBM model protein on assembly structure and the protein forming a bicontinuous assembly has been discussed in our previous work.^64,70^ The same effect is expected for the hydrophobic interactions of silk protein with its surroundings (surfaces, molecules). It is also known based on prior work that chain length in related silk-like proteins has a strong influence on assembly phases and the materials rising from them.^114^ The molecular level interactions changes due to the presence of ethanol could be used to tune the response. Indeed, previously, we have examined the effects of ethanol and urea as solvent additives on synthetic polyelectrolytes: ethanol depleted from the charged moieties but readily solvated the hydrophobic segments, promoting ion condensation that could also have an additional effect on protein response.^115^

**Figure 9:**
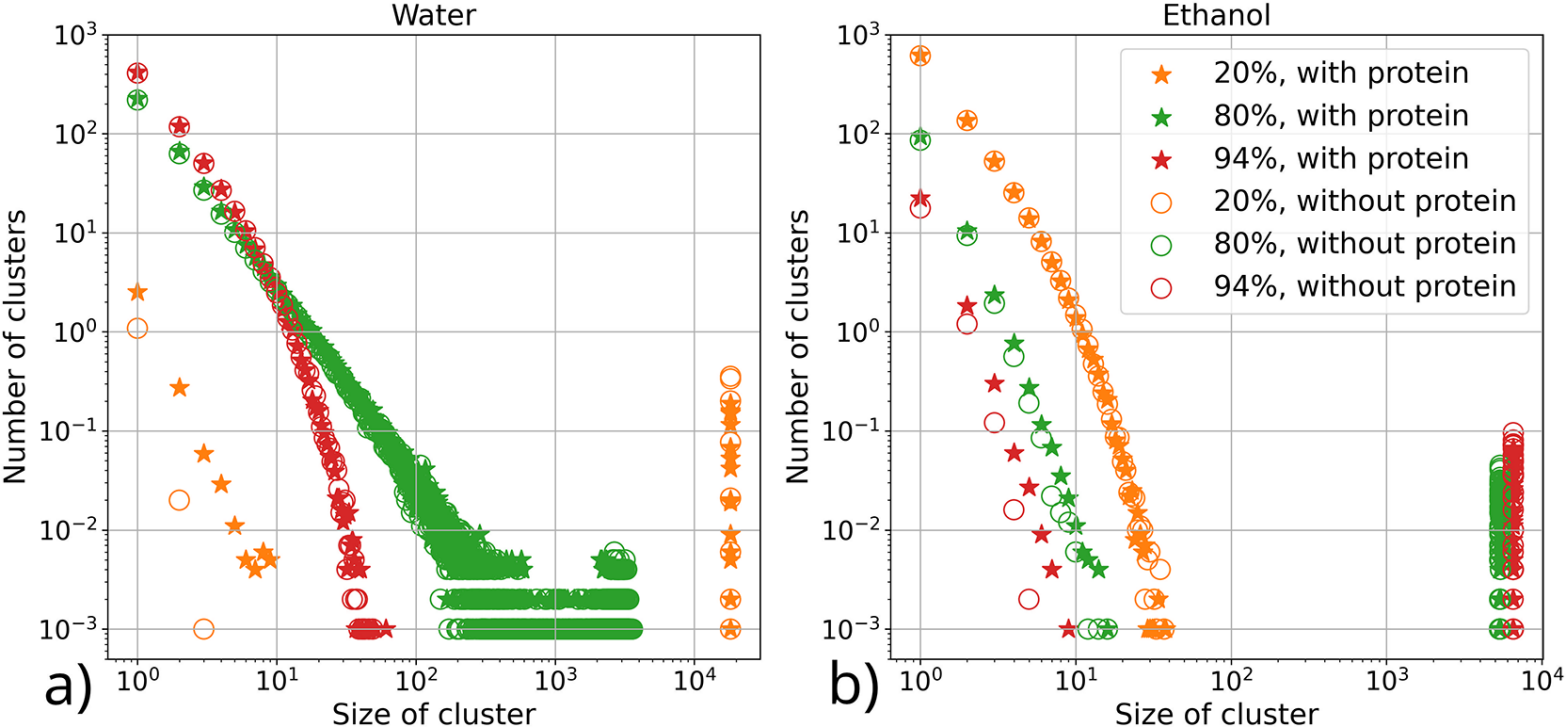
Distributions of cluster sizes formed by individual solvent components (water or ethanol) with and without protein in the system. Two solvent molecules (water–water or ethanol–ethanol) were considered to form a cluster if any of the atoms in the molecules were within 0.25 nm cutoff distance of each other. The distance is based on the hydrogen bond formation distance between the atoms of the molecules.

The microstructure of the ethanol/water mixture can be influenced by the local solvation preferences of the protein, i.e., the solvent distribution along the protein chain can affect not only the protein–protein interactions, but also the solvent characteristics. Figure 9 compares the distribution of water and ethanol clusters with and without protein in the simulation system. The difference in the data sets reveals how spidroin affects the microstructure of the ethanol/water mixture.

Data of Figure 9 at low concentrations of the solvent component, namely, the cluster distributions, are consistent with the findings for pure water/ethanol mixtures published earlier by Ghoufi et al.^59^ At a high concentration of a specific solvent component, almost all molecules of that component species in the system form one large cluster. This is due to the soft, purely distance-based criteria for determining the existence of a cluster (contact angle criteria between solvent molecules in the same cluster are not used). The presence of the silk protein in the system slightly changes the distribution of cluster sizes of the solution component with higher concentrations (water at a low concentration of ethanol, ethanol at a high concentration of ethanol). This is notable as the effect rises from the entire simulation box. However, as expected, solvent molecules adsorbed by the protein are not included in bulk clusters; instead, they form smaller clusters around the protein. This increases the number of small clusters (less than 10 molecules in size) and slightly decreases the size and number of large clusters in the solution when the protein is present. If the concentration of the solvent component is low, then it distributes as small clusters in the majority component. This distribution is not significantly affected by the presence of the protein.

## Conclusions

The molecular details of the effects of ethanol on spidroin protein secondary structure were studied using atomistic detail MD simulations supported by experimental characterization. Both approaches indicate a significant increase in the ordered structure when ethanol was added. Interestingly, in the experiments, the fraction of ordered structures in the ethanol/water mixtures was higher than the fraction of alanine-rich regions. This likely indicates that the proteins aggregate and form supramolecular structures when exposed to an ethanol-containing solvent. Beyond this, ethanol-induced a clear increase in ordered structures in the simulations, stabilizing existing α-helices and inducing the formation of additional turns. This has a direct significance on assembly characteristics and surface interactions of the proteins.

The findings identify the increase in intramolecular hydrogen bonding associated with the change in the secondary structure of the protein, but also with the aggregation of the protein molecules at high concentrations of ethanol, which we observed experimentally. Consideration of the modeling findings allows deducing that the aggregation is likely to occur due to the formation of H-bonds between protein molecules with the partial transition of α-helices into β-sheets connecting different protein molecules. Such a structural response has been shown previously for silk fibers regenerated by alcohol treatment.^104^ Here, we see the molecular mechanism of the phenomenon.

The work also allowed pinpointing the effects to individual amino acids, which provides a sequence-dependent tuning means for protein systems and the strength of their ethanol effects. Such amino acid level information aids in making guided decisions regarding designing molecular components for biomaterials with advanced materials response. In particular, glycine provides conformational freedom for the change in the secondary structure, while the shift in the balance between intermolecular and intramolecular interactions of polar amino acids (glutamine and serine) acts as the main driving force of the conformational changes induced by ethanol addition. The change in the proportion of these amino acids in the protein amino acid sequence should most significantly tune the alcohol effect on the secondary structure.

We also identified the molecular level solvation dependencies of the protein with the different solvent species. Namely, water solvated the hydrogen bonding moieties, while ethanol condenses also at hydrophobic chain segments, providing a more even ethanol distribution along the protein molecule. The ethanol molecules located along α-helices prevent hydrophobic interactions between them and can also affect the hydrophobic interactions of the protein with the surrounding. The molecular level solvation also influences, e.g., effect of ions and mechanical properties of the assemblies, see e.g. ref (115).

The results demonstrate that ethanol provides a convenient way to control the properties of silk materials via the spidroin secondary structure. For example, an increase in the ordering of the protein structure leads to an improvement in the thermal and degradative stability of silk and the various properties of the material based on it.^23,24,116^ The distribution of the β-sheet domains in the silk fibroin determines its mechanical properties.^22^ Moreover, the increase in the β-sheet content increases the transparency of silk-based films.^23^ Changes in the secondary structure of the protein can also affect the interfacial interactions in composite materials based on proteins.^36,69^ Overall, the significance of the present work is that the molecular level knowledge of the influence of ethanol can be used for systematic tuning of the protein systems, for spidroins, but also for other intrinsically disordered proteins that adapt their secondary structure to the solvent additive.

## Data Availability

Data associated with the manuscript, including simulation input files, number data for the figures, and analysis scripts are available at https://doi.org/10.23729/818b2e21-eb48-4571-817a-c153216cd7b8.

## Supporting Information Available

Stepwise simulation protocol; Time dependencies of structural characteristics of the investigated systems; High tension spectra from experimental CD measurements; Photograph of fully aggregated protein in 92.7% ethanol solution; Size distribution of protein particles by number of detected particles measured by DLS; Calculated numerical results from the DLS characterization; Light microscopy images of protein clusters forming in different solvents; Distributions of the secondary structures of the silk protein in water; The averaged values of the secondary structure ratio of the silk protein; Probability density distribution of secondary structures of the spidroin in water; Probability density distribution of the number of intramolecular and intermolecular protein H-bonds per one amino acid for each type of amino acid; Estimation of the solvent distribution along the protein chain; Radial distribution functions of the center of mass of water molecules, the neighboring amino acids, and the ethanol molecules around the center of mass of each type of amino acid; Positions of the first minima of these RDFs; Detailed distribution of the solvent molecules and protein residues along the protein chain (PDF)
