# Supplementary material for: Spidroins under the Influence of Alcohol: Effect of Ethanol on Secondary Structure and Molecular Level Solvation of Silk-Like Proteins
Source: Biomacromolecules. 2023 Nov 29;24(12):5638–53. doi: 10.1021/acs.biomac.3c00637 (PMC10716855; doi:10.1021/acs.biomac.3c00637)
Supplement: Supplementary file 1 — bm3c00637_si_001.pdf [file bm3c00637_si_001.pdf]

Supporting information

Spidroins under the influence of alcohol: effect of ethanol on secondary structure and molecular level solvation of silk-like proteins

Dmitry A. Tolmachev[1,3], Maaria Malkamäki[2,3], Markus B. Linder[2,3], and Maria Sammalkorpi[1,2,3]**

1 Department of Chemistry and Materials Science, Aalto University, P.O. Box 16100, FI-00076 Aalto, Finland

2 Department of Bioproducts and Biosystems, Aalto University, P.O. Box 16100, FI-00076 Aalto, Finland

3 Academy of Finland Center of Excellence in Life-Inspired Hybrid Materials (LIBER), Aalto University, P.O. Box 16100, FI-00076 Aalto, Finland

Supporting information consist of

- Stepwise simulation protocol.
- Figure S1. Time dependencies of structural characteristics of the investigated systems used for estimation of equilibration time.
- Figure S2. High tension spectra from experimental circular dichroism (CD) measurements as a quality measure of the collected data.
- Figure S3. Photograph of fully aggregated protein in 92.7 % ethanol solution at approximately 1 hour after sample preparation.
- Figure S4. Size distribution of protein particles by number of detected particles measured by Dynamic Light Scattering (DLS) in different solvents.
- Table S1. Calculated numerical results from the DLS characterization.
- Figure S5. Light microscopy images of protein clusters forming in different solvents.
- Figure S6. Distributions of the secondary structures of the silk protein in water.
- Table S2. The averaged values of the secondary structure ratio of the silk protein.
- Figure S7. Probability density distribution of secondary structures of the spidroin in water.
- Figure S8. Probability density distribution of the number of intramolecular and intermolecular protein H-bonds per one amino acid for each type of amino acid.
- Figure S9. Radial distribution functions of the center of mass of water molecules around the center of mass of each type of amino acid.
- Figure S10. Radial distribution functions of the center of mass of neighbor amino acids around the center of mass of each type of amino acid.
- Figure S11. Radial distribution functions of the center of mass of ethanol molecules around the center of mass of each type of amino acid.
- Table S3. Positions of the first minima on RDFs of surrounding molecules around amino acids.
- Figures S12-S13. The detailed distribution of the solvent molecules and protein residues along the protein chain.
- Figure S14. Radial distribution functions (RDF) of alanines in different helical regions relative to each other.

Stepwise simulation protocol.

1. Initial configuration of the model spidroin was adapted from the study of Batys *et al.*[1]. The initial, extended spidroin conformation presented in Figure 1 of Batys *et al.*[1] was used as the initial configuration.

2. Solvation of the spidroin model by water in a cubic simulation box 9.0 x 9.0 x 9.0 nm^3 in size was performed using **gmx solvate**. The solvation water configurations used for the solvation are part of the standard Gromacs 2022.3 software[2,3] distribution. The box of water for the solvation procedure was 1.86 x 1.86 x 1.86 nm^3 and it contained 864 TIP4P-ew water molecules.

3. The spidroin was simulated in pure water solution for 100 ns in the NPT ensemble. This resulted in the initial relaxation of both the spidroin and the simulation box size.

4. 0.149 M NaCl ions (for the water solvation system) and varying concentrations of ethanol (for the water/ethanol mixture solvation systems) were next added:

   4.1. The 0.149 M NaCl as ions was added by replacing randomly chosen water molecules using **gmx genion** to the system with only water as solvent, to match the experimental system.

   4.2. Ethanol was introduced to the systems by the following procedure:

   - An equilibrated bulk of pure ethanol was created by simulating a system of 857 ethanol molecules. This ethanol solvation box was created by random insertion of ethanol molecules followed by equilibration in NPT ensemble following the parameters of the final production run during 1 ns. The final size of the ethanol solvation box was 4.39 x 4.39 x 4.39 nm^3 corresponding to a final density of the solvation box 778.82+/-12.64 kg/m^3. The density is consistent with the experimental value within the error (789.24 kg/m^3 [4]).

   - After this, the water molecules that were not in the water shell around the protein at the completion of the 100 ns initial equilibration were removed. The number of removed water molecules (and as a result the final ethanol concentration) was controlled by varying the radius of the water shell.

   - The ethanol molecules were added by the **gmx solvate** tool into the box 9.0 x 9.0 x 9.0 nm^3 in size.

   - To initialize the simulation, energy minimization and preliminary MD simulation with a very short 0.0005 ps simulation step and Berendsen barostat and thermostat, with time constants 0.1 and 0.2 ps, respectively, was performed for 1 ps. This was to stabilize the ethanol for further equilibration.

5. Equilibration run with 0.002 ps simulation step using the Nosé-Hoover thermostat[5,6] and the Parrinello-Rahman isotropic barostat[7] (NPT ensemble) for 300 ns was performed

6. Production run for 700 ns was performed in the NPT ensemble.

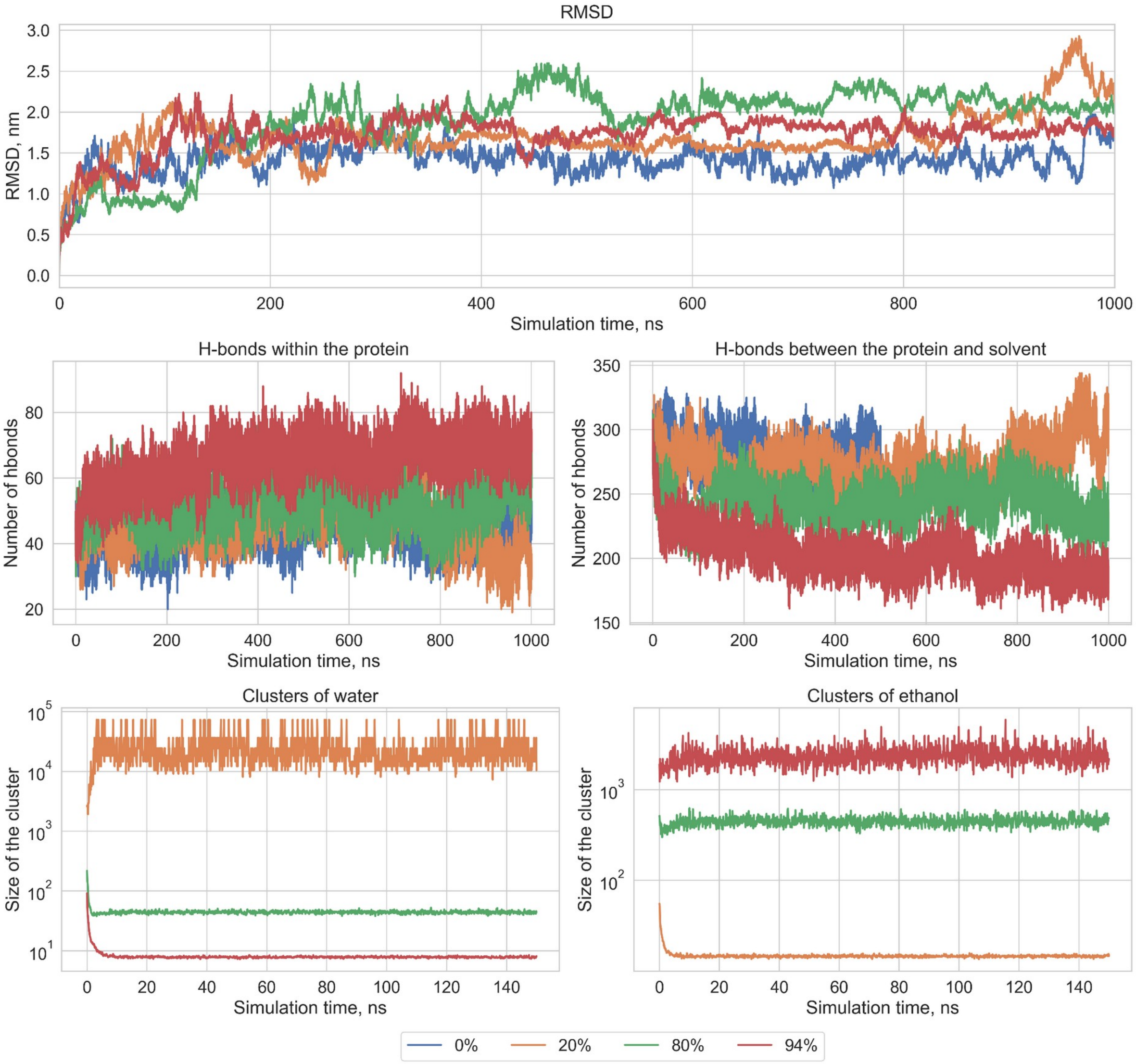

Figure S1. Time dependencies of structural characteristics of the investigated systems. The data is used for the estimation of equilibration time. The presented data include Root-mean-square deviation (RMSD) of protein, the number of H-bonds of protein with itself and with the solvent, and the average size of the water and ethanol clusters. Based on this data, the last 700 ns were chosen as the production run.

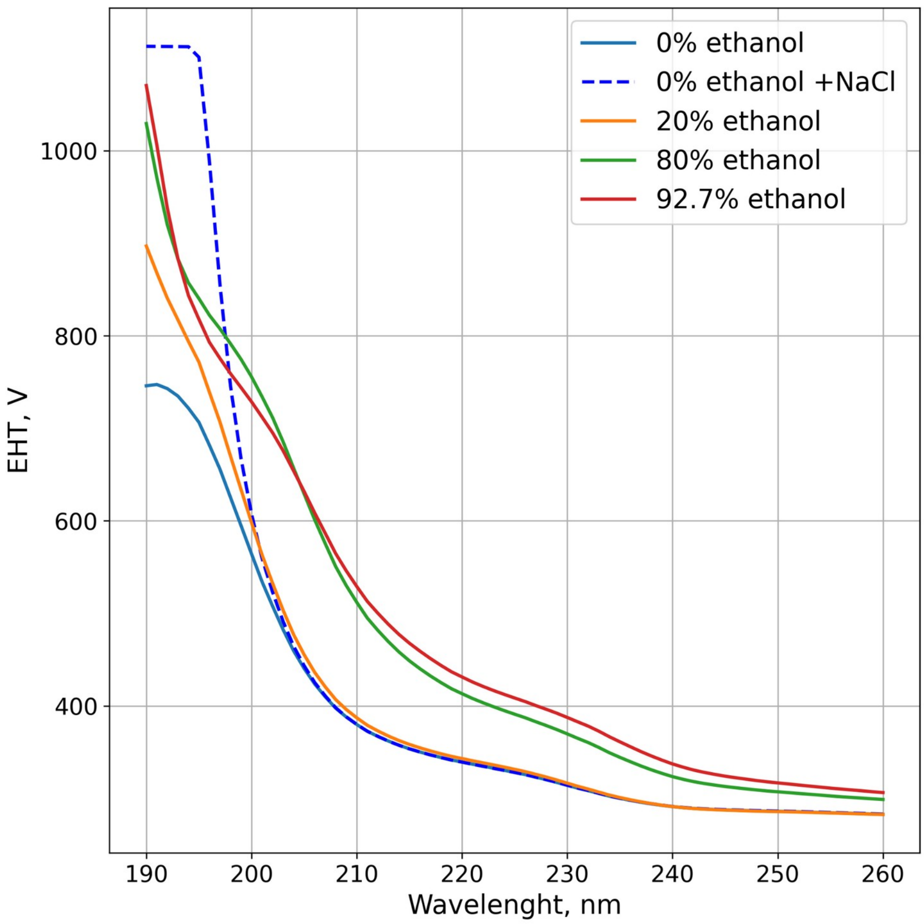

Figure S2. High tension spectra from experimental CD measurements as a quality measure of the collected data. The blue line represents the protein in water, and the dashed blue spectrum represents the protein in water with the presence of salt (NaCl), orange, green, and red spectra represent protein in ethanol-water mixtures containing 20, 80, and 92.7% of ethanol (v/v), respectively. High tension values below 600 V are considered as good-quality data.

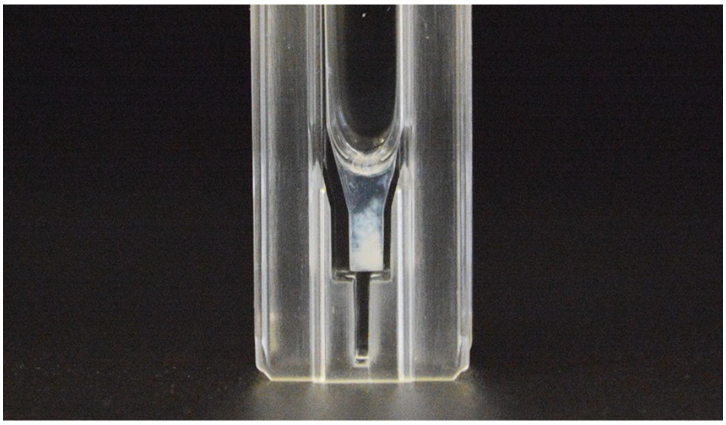

Figure S3. Photograph of fully aggregated protein in 92.7 % ethanol solution. The photograph corresponds to time approximately 1 hour after sample preparation. Over an extended time duration, the white aggregates sediment at the bottom. The rest of the solution is clear and transparent then.

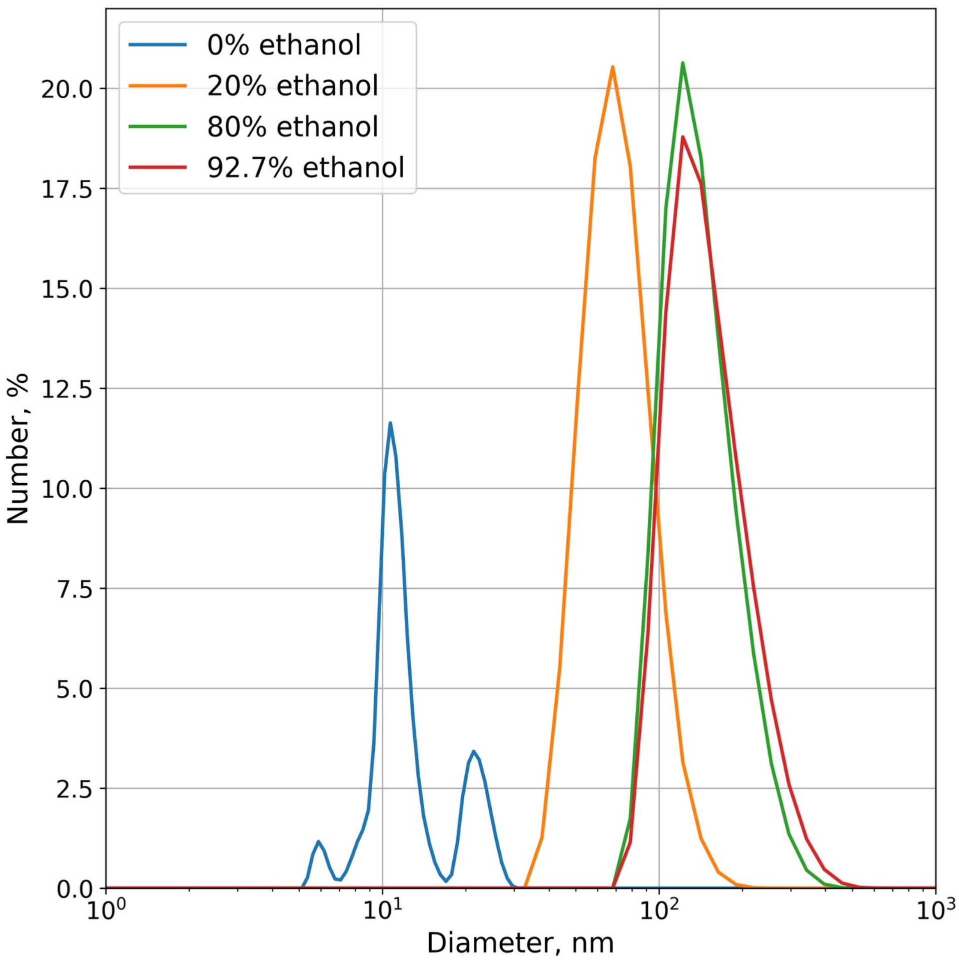

Figure S4. Distribution of protein particle sizes by number of detected particles measured by Dynamic Light Scattering (DLS) in the different solvent compositions. The presented distributions are the average distributions calculated from replicate runs of each sample.

The DLS results in terms of numerically calculated values are collected in Table S1. The data includes both intensity and number distribution-based values of the particle diameters. Notably, intensity is the direct measurement quantity of the DLS measurement and can be therefore considered more reliable. The intensity-based values are thus primarily used for the analysis of 20, 80, and 92.7 % ethanol samples. For the 0% ethanol sample, intensity could not be used as a reliable result because the characterized sample contained a single larger particle, which dominated the intensity distribution data as the actual particles were 10-fold smaller than in other samples, as revealed by the number distribution based data. In 0% ethanol samples, the number distribution gives much more reliable results although the data is not in primary format anymore. This data also does not show significant contributions due to larger particles (Figure S4) concluding that indeed a rare large particle was dominating the intensity signal. The presence of the larger particle in this sample also increased the DLS-measured polydispersity index to a high value. In general, this indicates worse sample quality. However, because the results from multiple samples in different concentrations of silk in water were similar, the results are considered reliable. The distributions of the number of particles are also in good agreement with the intensity distributions for 20, 80, and 92.7 % ethanol samples, showing only slight variation in the

average values. This indicates that the distribution of the number of particles from DLS can be used reliably with these samples. Derived count rate in kilo counts per second (kcps) gives an indication of sample concentration, or here, the number of detected particles. The mean count rate of sample measurements is kept relatively constant for each sample with the help of attenuators to protect the detector which is sensitive. The derived count rate is the theoretical value calculated from the mean count rate by removing the effect of attenuators and it represents the situation where 100% of the laser signal would have been detected by the detector.

Table S1. Calculated numerical results from the DLS characterization.

Ethanol concentration (v/v), %	Z-ave from intensity distribution, nm	Number distribution average, nm	Polydispersity index	Derived count rate, kcps
0	259.8	13.1	0.85	1313
20	92.1	73.1	0.173	1443
80	174.1	145.4	0.070	52 323
92.7	193.4	156.8	0.091	149 228

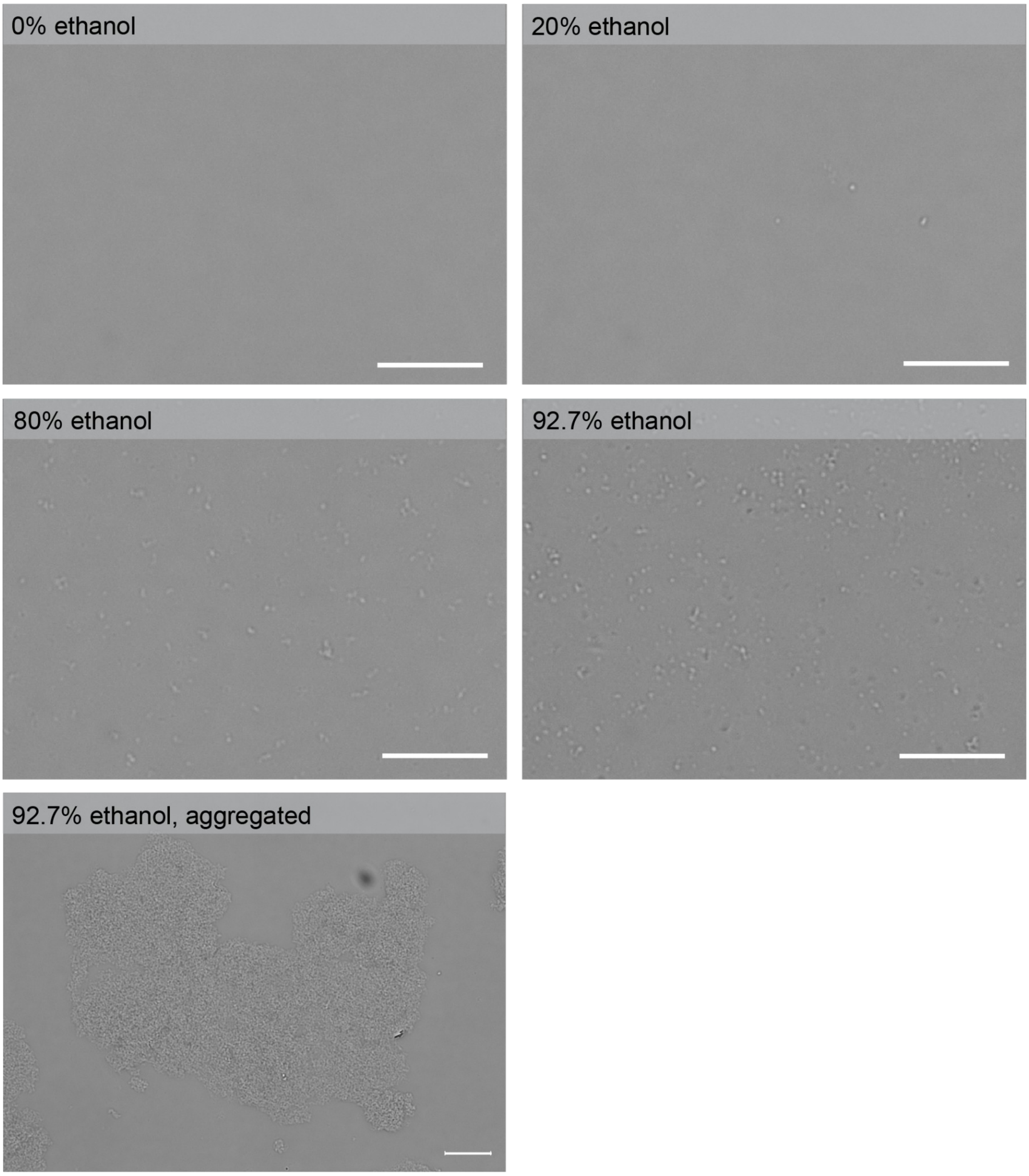

Figure S5. Light microscopy images of protein clusters forming in different solvents. Scale bar is 20 µm. Images are taken directly after sample mixtures were prepared, except in the case of the aggregated sample. The aggregated image corresponds to a sample that is over 1 hour old at the moment of imaging.

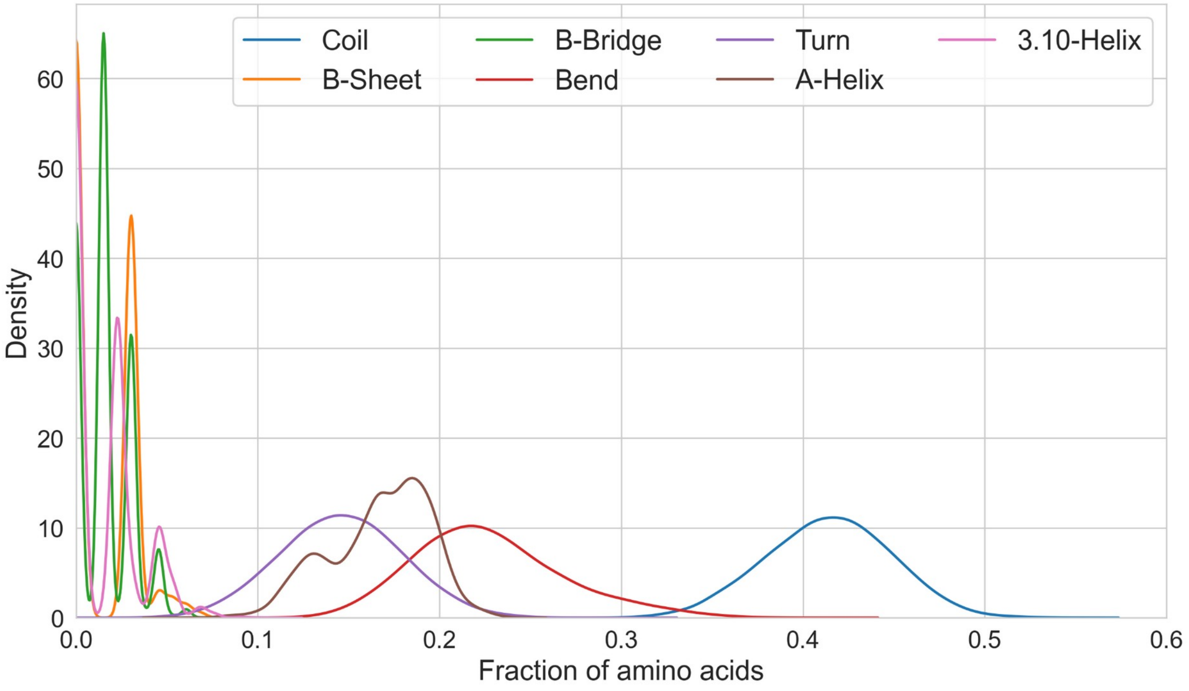

Figure S6. Probability density distribution of secondary structures of the spidroin in water. Distribution plotted using kernel density estimation. The summed distributions of 3.10- and α-helices; turns, β-sheets, and β-bridges are shown in Figure 3 of the main manuscript.

Table S2. The averaged values of the secondary structure ratios calculated based on the distributions shown in Figure 4, panels a, c, d, e, g in the main manuscript.

Concentration of ethanol (v/v), %	3.10- and α-helices	Turns, β-sheets, and β-bridges	Coil	Bend
0	0.18 ± 0.03	0.18 ± 0.04	0.41 ± 0.04	0.23 ± 0.04
20	0.15 ± 0.04	0.18 ± 0.05	0.43 ± 0.04	0.24 ± 0.06
80	0.18 ± 0.03	0.21 ± 0.05	0.38 ± 0.04	0.23 ± 0.04
94	0.20 ± 0.02	0.26 ± 0.04	0.33 ± 0.03	0.21 ± 0.03
Difference between 0% and 94% systems	0.02 ±0.04	0.08 ± 0.05	-0.08 ± 0.05	-0.02 ± 0.05

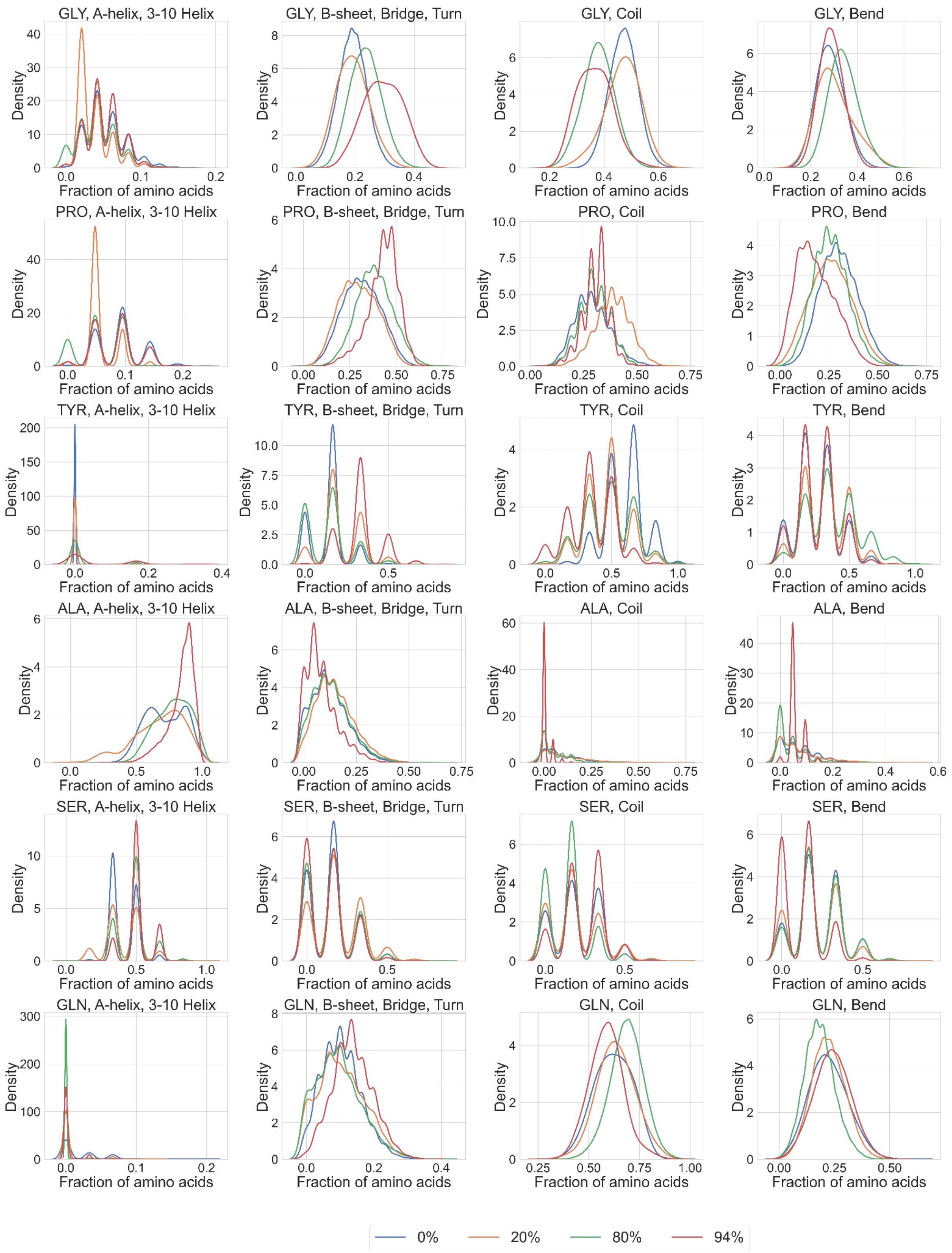

Figure S7. Probability density distribution of the secondary structures for each type of amino acid in the silk protein. Distribution plotted using kernel density estimation. The averaged values are shown in Figure 4, panels b, d, f, h, in the main manuscript.

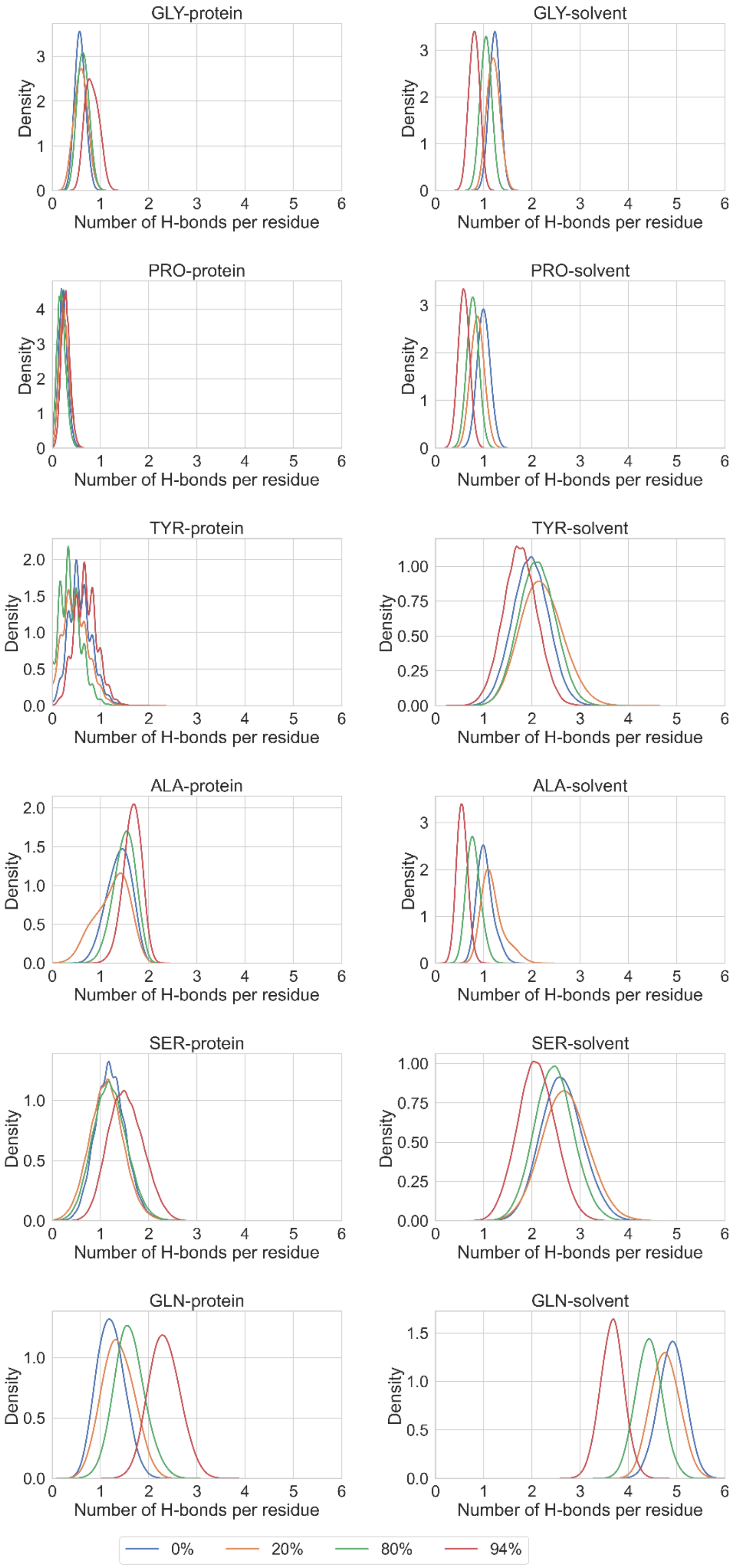

Figure S8. Probability density distribution of the number of intramolecular (right) and intermolecular (left) protein H-bonds per amino acid for each type of amino acid. Distribution plotted using kernel density estimation. The summed distribution for the protein and averaged values are shown in Figure 5 b, d.

Description of estimation of the solvent distribution along the protein chain.

To analyze the distribution of the surrounding molecules along the protein chain, the number of molecules in a sphere around the center of mass (COM) of each amino acid in the protein sequence was determined. The size of the analysis sphere was dependent on the amino acid type and determined based on radial distribution functions (RDFs) of the COM of the solvent molecules and the neighboring amino acids around the COM of each amino acid. Examples of RDFs for the analyzed systems are shown in Figures S6-S8.

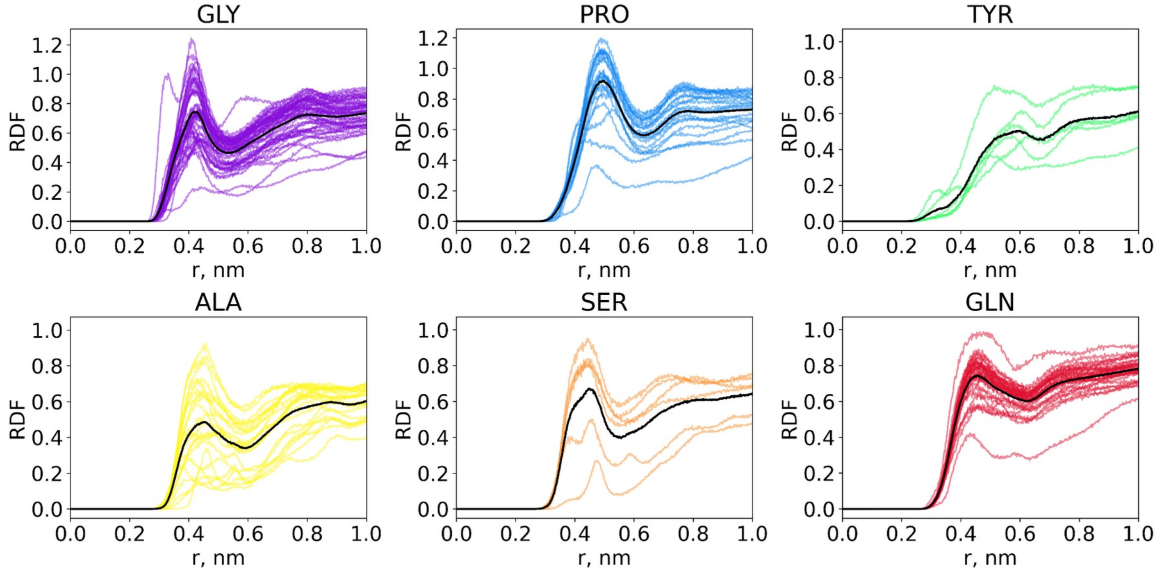

Figure S9. Radial distribution functions calculated between the center of mass of any water molecule and the center of mass of each amino acid. The colored lines represent RDFs for each amino acid, and the black lines show the average RDF. Data for the system with 0% added ethanol is presented.

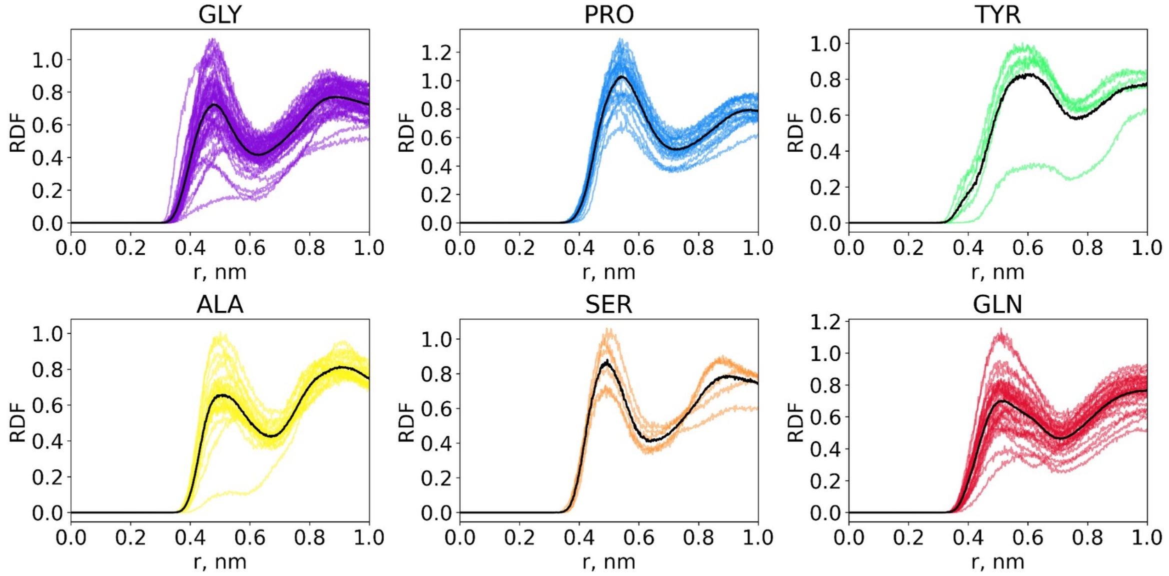

Figure S10. Radial distribution functions calculated between the center of mass of any ethanol molecule and the center of mass of each type of amino acid. The colored lines represent RDFs for each amino acid, and the black lines show the average RDF. Data for the system with 94% added ethanol is presented.

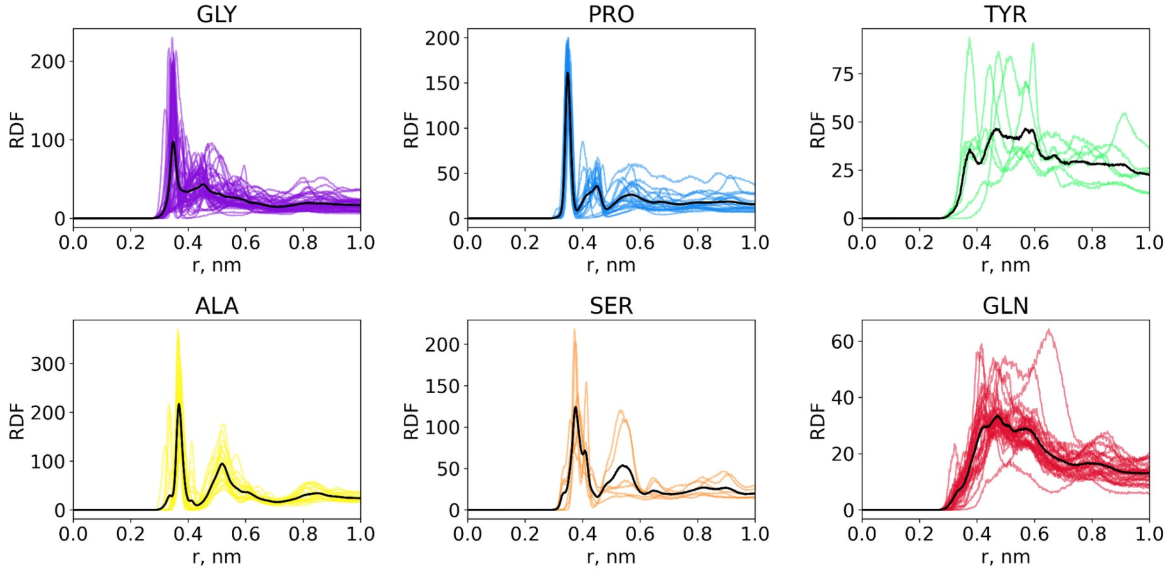

Figure S11. Radial distribution functions calculated between the centers of mass of the neighboring amino acids and the specified amino acid. The colored lines represent RDFs for each amino acid, and the black lines show the average RDF. Data for the system with 0% added ethanol is presented.

The minimum after the main peak (the nearest neighbor peak) was used as the size of the analysis sphere. The radii of spheres are collected in Table S2. The position of minima differs for the different types of molecules. The position corresponding to the minima on ethanol RDF was chosen as the radius for the spheres as the value is larger. The number of molecules in the sphere (shown in Figures S9-S10) was calculated as a cumulative number at this distance based on the integration of the RDFs. Density was calculated based on this number and the volume of the spheres. The results are shown in Figure 6.

Table S3. Positions of the first minima on RDFs of the solvent molecules or the neighboring amino acids around each amino acid. The distances for ethanol were chosen as a radius for the spheres for analysis shown in Figures 6 (main manuscript), S9 and S10.

	GLY, nm	PRO, nm	TYR, nm	ALA, nm	SER, nm	GLN, nm
ethanol	0.628	0.728	0.758	0.672	0.640	0.708
water	0.538	0.632	0.640	0.594	0.556	0.628
protein	0.392	0.380	0.404	0.430	0.452	0.598

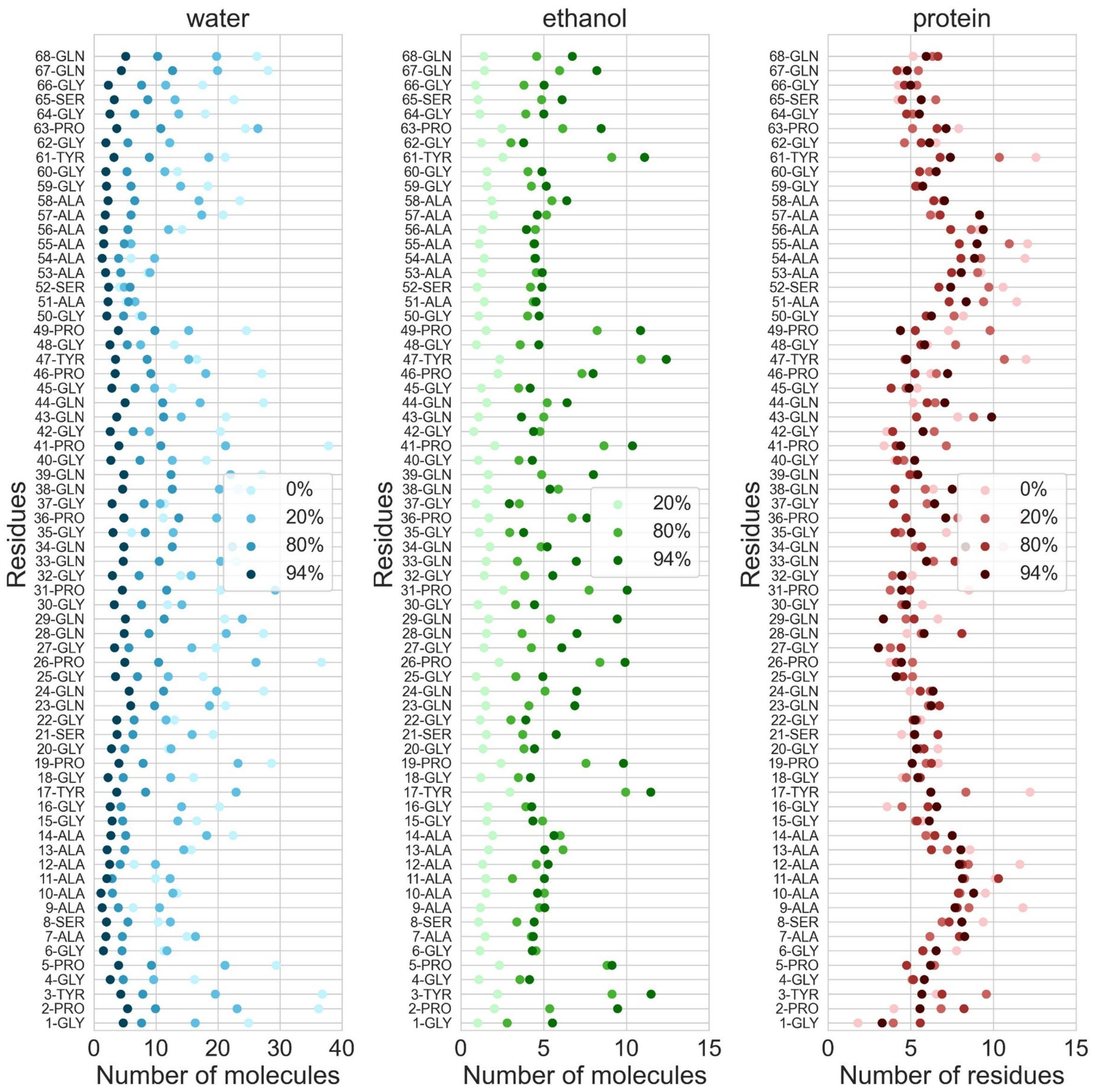

Figure S12. Detailed distribution of the solvent molecules and protein residues along the protein chain. Data corresponding to amino acids 1 to 68 presented.

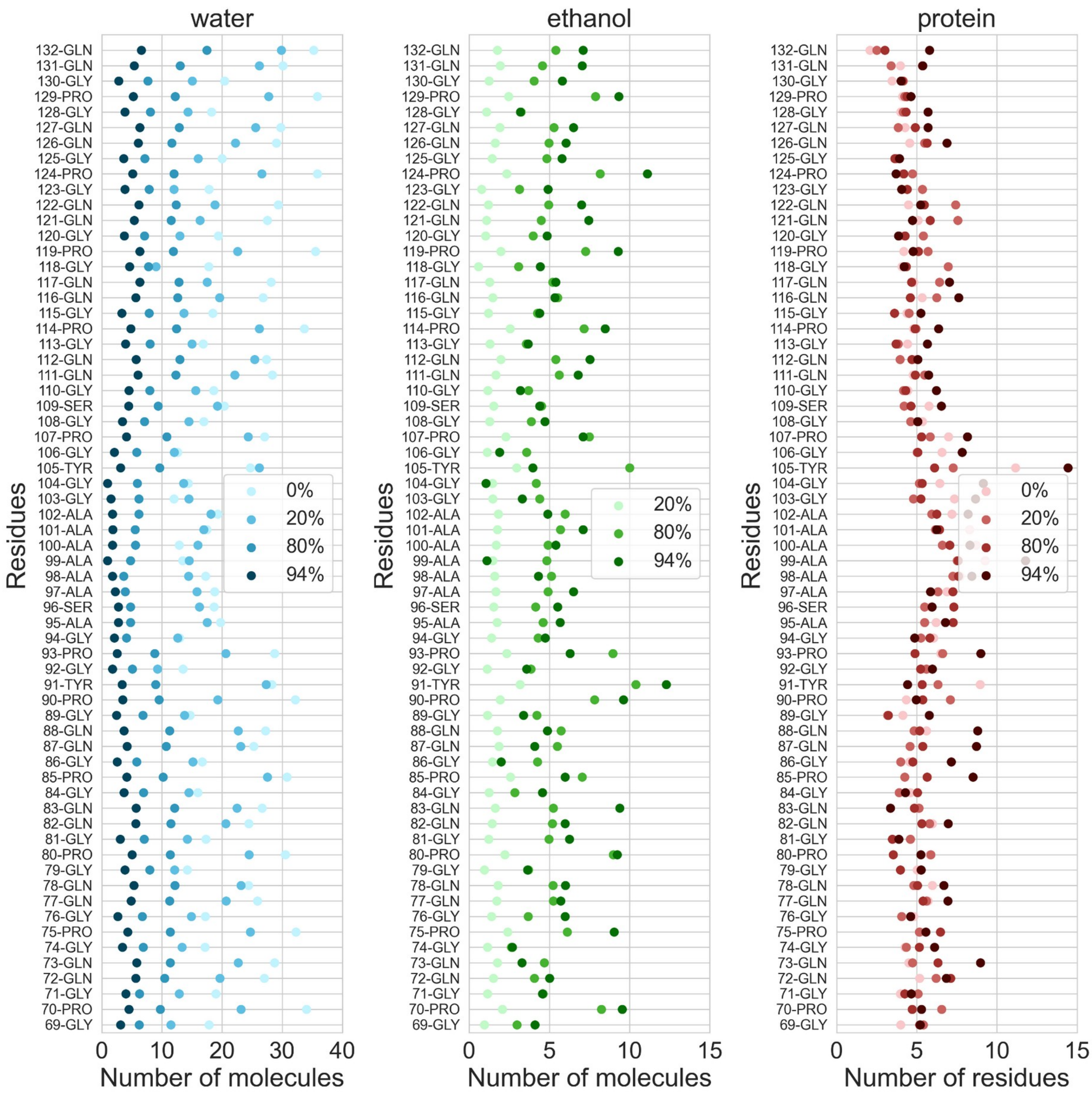

Figure S13. Detailed distribution of the solvent molecules and protein residues along the protein chain. Data corresponding to amino acids 69 to 132 presented.

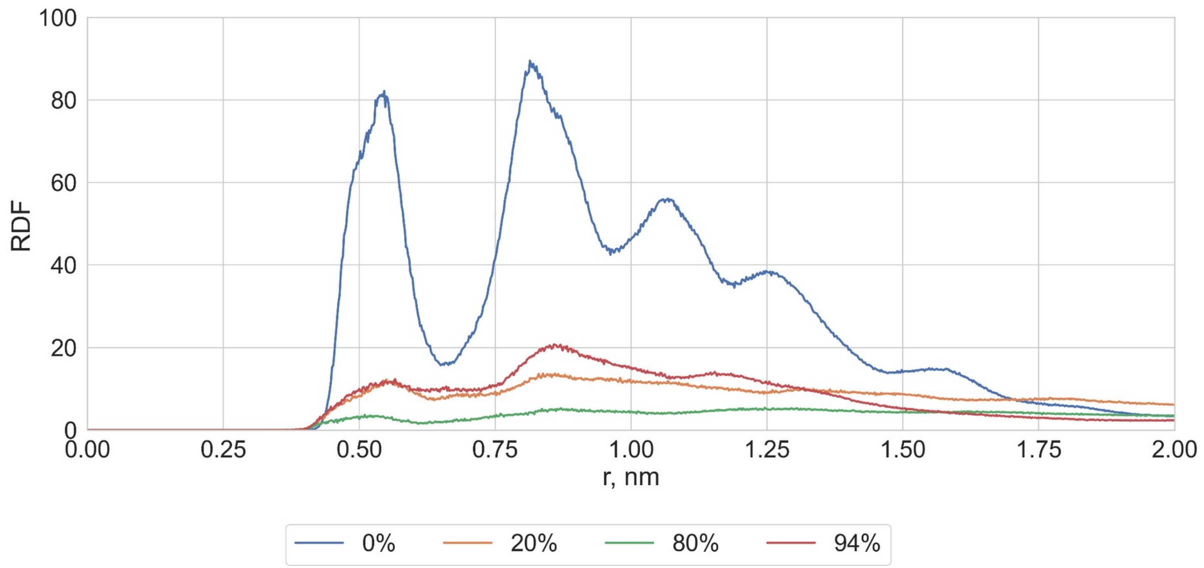

Figure S14. Radial distribution functions (RDF) of alanine residues in different helical regions. The calculation uses each alanine residue center of mass vs the other alanine residues centers of mass, i.e. relative to each other. In water, RDF is much higher. This indicates strong hydrophobic interaction between α-helices in the spidroin.

References:

1. Batys, P. et al. Self-Assembly of Silk-like Protein into Nanoscale Bicontinuous Networks under Phase-Separation Conditions. Biomacromolecules 22, 690–700 (2021).

2. Abraham, M. J. et al. GROMACS: High performance molecular simulations through multi-level parallelism from laptops to supercomputers. SoftwareX 1-2, 19–25 (2015).

3. Páll, S., Abraham, M. J., Kutzner, C., Hess, B. & Lindahl, E. Tackling Exascale Software Challenges in Molecular Dynamics Simulations with GROMACS. in Solving Software Challenges for Exascale 3–27 (Springer International Publishing, 2015).

4. Alcoholometry: 'International Alcoholometric Tables'. (Technology Reports Centre, Department of Industry for the Department of Prices and Consumer Protection, 1975).

5. Hoover, W. G. Canonical dynamics: Equilibrium phase-space distributions. Phys. Rev. A Gen. Phys. 31, 1695–1697 (1985).

6. Nosé, S. A unified formulation of the constant temperature molecular dynamics methods. The Journal of Chemical Physics vol. 81 511–519 Preprint at https://doi.org/10.1063/1.447334 (1984).

7. Parrinello, M. & Rahman, A. Polymorphic transitions in single crystals: A new molecular dynamics method. J. Appl. Phys. 52, 7182–7190 (1981).
